# Reflux of Endoplasmic Reticulum proteins to the cytosol inactivates tumor suppressors

**DOI:** 10.15252/embr.202051412

**Published:** 2021-03-12

**Authors:** Daria Sicari, Federica G Centonze, Raphael Pineau, Pierre‐Jean Le Reste, Luc Negroni, Sophie Chat, M Aiman Mohtar, Daniel Thomas, Reynald Gillet, Ted Hupp, Eric Chevet, Aeid Igbaria

**Affiliations:** ^1^ Inserm U1242 University of Rennes Rennes France; ^2^ Centre de lutte contre le cancer Eugène Marquis Rennes France; ^3^ Neurosurgery Department University Hospital of Rennes Rennes France; ^4^ Institut de Génétique et de Biologie Moléculaire et Cellulaire Illkirch France; ^5^ UMR7104 Centre National de la Recherche Scientifique Illkirch France; ^6^ U1258 Institut National de la Santé et de la Recherche Médicale Illkirch France; ^7^ Université de Strasbourg Illkirch France; ^8^ CNRS Institut de Génétique et Développement de Rennes (IGDR) UMR6290 Univ. Rennes Rennes France; ^9^ Edinburgh Cancer Research Centre at the Institute of Genetics and Molecular Medicine Edinburgh University Edinburgh UK; ^10^ International Centre for Cancer Vaccine Science Gdansk Poland; ^11^ Department of Life Sciences Ben‐Gurion University of the Negev Beer Sheva Israel; ^12^ Present address: IFOM Fondazione Istituto FIRC di Oncologia Molecolare Milan Italy; ^13^ Present address: UKM Medical Molecular Biology Institute (UMBI) Universiti Kebangsaan Malaysia Kuala Lumpur Malaysia

**Keywords:** cancer, endoplasmic reticulum, ER stress, ERAD, reflux, Cancer, Organelles, Translation & Protein Quality

## Abstract

In the past decades, many studies reported the presence of endoplasmic reticulum (ER)‐resident proteins in the cytosol. However, the mechanisms by which these proteins relocate and whether they exert cytosolic functions remain unknown. We find that a subset of ER luminal proteins accumulates in the cytosol of glioblastoma cells isolated from mouse and human tumors. In cultured cells, ER protein reflux to the cytosol occurs upon ER proteostasis perturbation. Using the ER luminal protein anterior gradient 2 (AGR2) as a proof of concept, we tested whether the refluxed proteins gain new functions in the cytosol. We find that refluxed, cytosolic AGR2 binds and inhibits the tumor suppressor p53. These data suggest that ER reflux constitutes an ER surveillance mechanism to relieve the ER from its contents upon stress, providing a selective advantage to tumor cells through gain‐of‐cytosolic functions—a phenomenon we name ER to Cytosol Signaling (ERCYS).

## Graphical abstract

Endoplasmic Reticulum (ER) stress in cancer cells causes a subset of ER proteins to escape to the cytosol where they bind and inhibit key signaling pathways to increase cancer cell fitness.

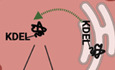

## Introduction

The endoplasmic reticulum (ER) is the gateway to the secretory pathway thus maintaining the communication between the cell’s intracellular space and extracellular environment. In addition, the ER is a sensing organelle that coordinates many stress signaling pathways (Higa & Chevet, 2012; Alexia *et al,* 2013; Hetz *et al,* 2015). Secretory and transmembrane proteins translocate into the ER through different translocation/membrane insertion molecular machines including the Sec61 channel. The ER is crowded with molecular chaperones and foldases that ensure these proteins’ productive folding followed by their export en‐route to their final destination (Rapoport, 2007). Diverse perturbations compromise the folding and maturation of secretory proteins in the ER thereby causing ER stress. To ensure productive folding, cells have also evolved various ER quality control (ERQC) systems allowing for further folding rounds (Adams *et al,* 2019) and their degradation in the cytosol by a process termed ER‐associated degradation (ERAD) (Travers *et al,* 2000; Rutkowski *et al,* 2006; Vembar & Brodsky, 2008). In addition to ERQC and ERAD, a pre‐emptive quality control (pre‐QC) mechanism was also described that averts protein entry into the secretory pathway under protein‐folding stress resulting in their proteasomal degradation in the cytosol (Kang *et al,* 2006). If these quality control systems are overwhelmed, ER stress activates a signaling pathway called the unfolded protein response (UPR) that aims at restoring ER homeostasis. However, if the UPR adaptive function fails, cell death programs are activated (Almanza *et al,* 2019). The UPR is transduced by three transmembrane proteins (PERK, ATF6α and IRE1α) that sense and monitor the protein‐folding status of the ER through their luminal domains and transmit signals to the rest of the cell through their cytosolic domain (Almanza *et al,* 2019).

In the past three decades, a subset of ER‐resident proteins was reported to accumulate in the cytosol. This was observed in several human diseases including cancer and degenerative diseases (Turano *et al,* 2002; Afshar *et al,* 2005; Tarr *et al,* 2010; Galligan & Petersen, 2012; Kanekura *et al,* 2015; Wiersma *et al,* 2015; Shim *et al,* 2018). The localization of proteins identified as ER‐resident to other cellular compartments has been extensively reported for instance for members of the protein disulfide isomerase (PDI) family, for GRP78/BiP or for calreticulin (Turano *et al,* 2002; Afshar *et al,* 2005; Tarr *et al,* 2010; Galligan & Petersen, 2012; Wiersma *et al,* 2015; Shim *et al,* 2018). Despite this recurring observation, the mechanisms by which ER‐resident proteins relocate in the cytosol and the potential functions of those proteins in this compartment remain unclear.

Recently, we showed that protein‐folding stress causes ER‐resident proteins to be refluxed to the cytosol in the yeast *Saccharomyces Cerevisiae* (Igbaria *et al,* 2019). This mechanism requires ER and cytosolic chaperones and co‐chaperones but is independent of ERAD and of protein degradation (Igbaria *et al,* 2019). Here, we found that ER stress‐mediated protein reflux is conserved in mammalian cells and in cancer cells isolated from human and murine tumors in which it aims at debulking the ER upon stress. Moreover, we found that this process to be constitutively active in tumor cells and lead to cytosolic gain‐of‐functions of the refluxed protein as inhibitor of tumor suppressors, thereby exhibiting pro‐oncogenic features.

## Results

### ER‐resident proteins are refluxed from the ER lumen to the cytosol in cancer cells isolated from human and murine GBM tumors

**Figure 1:**
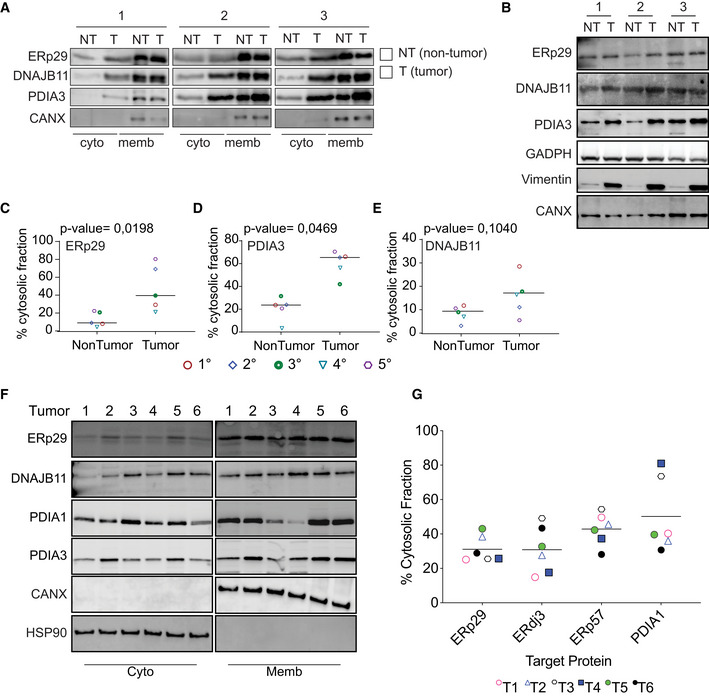
ER proteins are rerouted to the cytosol in human and mouse Glioblastoma (GBM) tumors ARepresentative Western blots after subcellular protein fractionation experiment in isolated murine‐derived non‐tumor (NT) and GBM tumor (T) tissues.BTotal levels of the ER‐luminal proteins tested from the cell lysate derived from tissues used in (A).C–EQuantification of the protein levels of ER luminal proteins in the cytosolic fraction as shown in (A). *n* = 5 biological replicates and the horizontal line represent the sample mean. Differences were analyzed by Unpaired Student’s *t*‐test using Prism 9 (GraphPad), except when otherwise indicated. *P*‐values < 0.05 were considered significant).F, GHuman‐derived GBM tumors were processed as in (A). Representative Western blot was performed (F), and the percentage of ER‐luminal protein cytosolic localization (G) were quantified. Data are the average from six different tumors. *N* = 6 Bars and error bars indicate mean

To study the role of ER protein reflux in tumors, we initially focused on Glioblastoma multiforme (GBM) in which the unfolded protein response (UPR) sustains tumor aggressiveness (Obacz *et al,* 2017). Mouse GBM cells (GL261) were grafted orthotopically in the brain of immunocompetent C57BL/6 mice and 30 days post‐injection, tumors were resected, dissociated, and isolated tumor cells subjected to subcellular fractionation using previously validated fractionation protocols (Holden & Horton, 2009). Immunoblot analysis of the digitonin fraction (enriched in cytosolic proteins) from freshly isolated tumor cells was compared with that of dissociated control tissue from the opposite hemisphere of the brain (non‐tumor). It revealed higher levels of select ER‐resident proteins in tumor than in the non‐tumor cells’ cytosolic fractions (Fig 1A‐1E and Appendix Fig S1A and B). Several ER‐resident proteins (ERp29/PDIA9 and ERp57/PDIA3) were enriched up to ∼ 70% in the cytosolic fraction compared with only ∼ 10% enrichment in non‐tumor controls. These results indicate that tumor cells are more prone to exhibit reflux of ER proteins to the cytosol than non‐tumoral cells. To rule out the possibility that ER stress in tumor cells may change the composition of the ER membrane thereby sensitizing it to digitonin treatment (detergent), we consequently used a digitonin‐free subcellular protein fractionation protocol (Lodish, 2000). Cells were disrupted using a 26‐gauge needle, and then, differential centrifugation was applied as shown in Appendix Fig S1C. After analyzing the cytosolic fractions, we observed results comparable to those obtained with the digitonin‐based protocol (Fig 1A and Appendix Fig S1D). We confirmed these findings in another GBM model, the human U87 cells orthotopically implanted in the brain of NSG mice (Appendix Fig S1E). These two different protocols further strengthen the notion that ER proteins do exit the ER to reach the cytosol more actively in tumor cells.

We next asked whether this phenomenon is also observed in human GBM samples freshly isolated from patients at surgery (Table S1). We tested the subcellular localization of ER‐resident proteins in freshly isolated human tumor cells. Tumor tissues were dissociated, and digitonin fractions tested for the presence of ER luminal proteins using immunoblotting. In the majority of tumors (80% of the tested tumors), ∼ 50% of the ER proteins evaluated were detected in the digitonin fraction including ERDJ3/DNAJB11 in its N‐glycosylated state (Fig 1F‐G and Appendix Fig S1F). Moreover, individual tumors exhibited heterogenous refluxed protein patterns, which might reflect inter‐tumor heterogeneity (Fig 1F and G). In both GBM tumors (human‐ or murine‐derived, N‐linked‐glycoproteins (such as ERDJ3/DNAJB11) were found in the digitonin fraction thus indicating that the refluxed proteins had been translocated into the ER and modified by N‐Linked glycosylation, before being refluxed to the cytosol (Appendix Fig S1G‐H).

These data indicate that in GBM, ER protein reflux might be selectively regulated by different factors such as tumor heterogeneity, genetic background, or activation status of UPR. These findings will certainly stimulate others to replicate and extend these data in other tumor models.

### ER stress mediates ER‐resident proteins reflux from the ER to the cytosol

**Figure 2:**
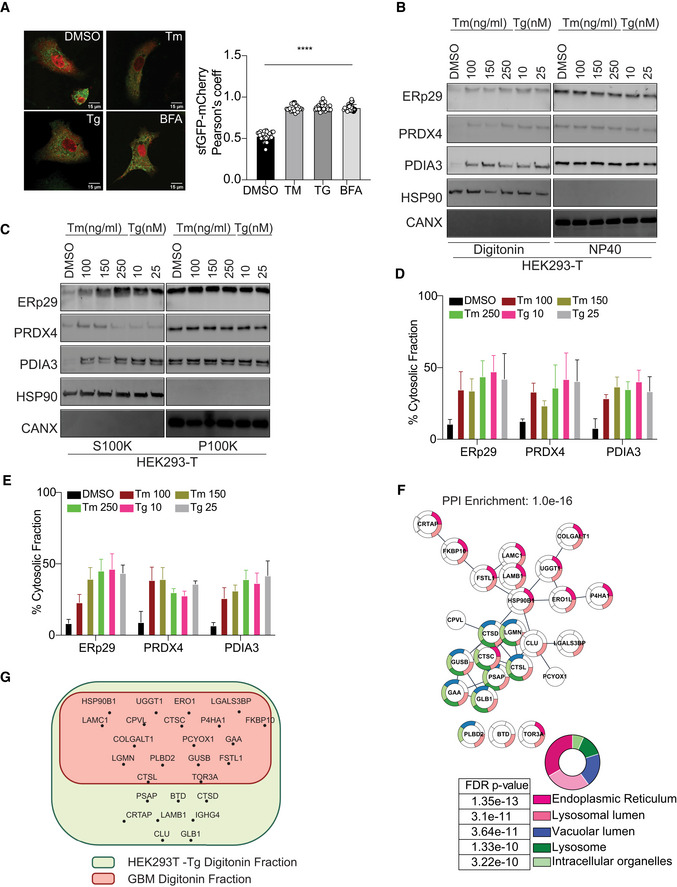
Pre‐existing, ER‐targeted mEOS3.2 and ER endogenous proteins reflux to the cytosol during ER stress ALeft: Representative images of cells were transfected with super‐folder GFP (sfGFP) treated with 250 ng/ml Tunicamycin (Tm), 50 nM Thapsigargin (Tg), and 250 ng/ml Brefeldin‐A (BFA) for 24 h. Right: Quantification of the microscopy images of cells expressing ER‐targeted sfGFP and the cytosolically localized mCherry. Data values are the mean ± SD of technical replicates (*n* = 10) from three independent experiments (****P < 0.0001). One‐way ANOVA was applied for the statistical analysis through the GraphPad Prism 9 software. Scale bar 15 μm.B, CSubcellular protein fractionation of several ER‐resident proteins in HEK293T cells treated with the indicated concentrations of Tm or Tg for 16 h using Digitonin (NP40 represents the membrane fraction extracted with NP40 Cell Lysis Buffer) (B) or differential centrifugation (C) protocols, representative Western blots are showed.D, EQuantification of the subcellular protein fractionation of several ER endogenous proteins in HEK293T cells treated with different concentrations of Tm and Tg for 16 h from panels (B and C), respectively. Biological triplicates, mean ± SD calculated using Prism 9 (GraphPad).FMass spec analysis of soluble ER‐targeted glycoproteins in HEK293T cells treated with Tg and analyzed as described in materials and methods. Biological triplicates and data analyses were carried using Cytoscape v3.8.0 for network representation (PMID: 14597658) with the Cytoscape Stringapp for enrichments (PMID: 30450911). Statistics were done using the default settings of the Cytoscape app.GMass spectrometry analysis of cytosolically located soluble ER glycoproteins in HEK293T cells treated with Tg compared with that found in human GBM tumor cells‐derived cytosols.

We next sought to identify factors regulating ER‐to‐cytosol protein reflux. Recently, we reported that ER luminal proteins were refluxed to the cytosol upon ER stress in the yeast *S. cerevisiae* (Igbaria *et al,* 2019; Lajoie & Snapp, 2020) in a chaperone‐mediated process (Igbaria *et al,* 2019). We tested whether ER stress/UPR activation—two factors that showed a correlation with protein reflux in *S. cerevisiae*—would also cause ER protein reflux in mammalian cells. As such we monitored the localization of an engineered ER‐targeted super‐folder GFP (ER‐sfGFP) using confocal microscopy, to follow the fate of ER‐sfGFP in living cells. The cells were also transfected with the cytosolically localized mCherry used as a cytosolic marker. Notably, cells treated with Tunicamycin (Tm), which perturbs protein folding by inhibiting N‐linked glycosylation, Thapsigargin (Tg) which inhibits the sarco‐endoplasmic reticulum Ca2 + ATPase or Brefeldin A (BFA) that prevents protein transport from the ER to the Golgi apparatus, showed enhanced colocalization of ER‐sfGFP with the cytosolic mCherry. The cytosolic colocalization of ER‐sfGFP/mCherry reached a maximum after 24hrs of treatment (Fig 2A and Appendix Fig S2A). To confirm that the ER‐resident proteins found in the cytosol after ER stress originated from the ER lumen, we engineered an ER‐targeted photoactivatable fluorescent protein (FP) called mEOS3.2 by adding an ER signal peptide and a KDEL ER‐retention sequence that discriminates newly synthesized proteins from the pre‐existing pool. Indeed, UV exposure shifts the excitation maxima of the mEOS3.2 from 488nm to 573nm, allowing detection of proteins synthesized before a UV pulse exposure (Appendix Fig S2B). Notably, after a UV pulse and Tm or DMSO treatments for 24 h, the mEOS3.2^573^ pool was mainly localized in the ER of the DMSO treated cells, but cells treated with Tm or BFA showed a significant fraction of mEOS3.2^573^ localized in the cytosol (Appendix Fig S2B). These results indicated that during stress pre‐existing and ER localized proteins are refluxed to the cytosol where they might exist in a folded, functional state.

We next tested whether endogenous ER‐resident proteins were also refluxed to the cytosol in cultured cells that were exposed to various ER stress inducers. Subcellular protein fractionation using minimal concentration of digitonin that results in proper separation of the different subcellular fractions was carried out in cells subjected to ER stress induced by Tm or Tg. This was followed by an analysis of the localization of different endogenous ER‐resident proteins including the soluble ERp29, PRDX4, PDIA3, and the integral protein calnexin (CANX). We found that soluble ER luminal proteins were enriched in the digitonin fraction up to 50‐55% (Fig 2B and D) but not calnexin, thus indicating that ER reflux could be exclusive for soluble proteins. We then compared those results to those obtained from the detergent‐free protocol (Lodish, 2000) using differential centrifugation, to rule out the possibility that ER stress inducers may alter the ER membrane properties toward digitonin. As shown in Fig 2C and E, we observed results similar to those obtained with the digitonin‐based protocol (Fig 2B and D). We further investigated this by examining the integrity of the ER membrane in those cells. We obtained pellets post‐digitonin fraction and after the 100,000xg centrifugation and treated them with proteinase K in the absence or presence of TritonX‐100. We reasoned that if the ER membrane is damaged/ruptured due to digitonin or differential centrifugation protocols, ER luminal proteins should be sensitive to proteinase‐K‐mediated proteolysis. As shown in Appendix Fig S2C and D, while proteinase K was active toward the cytosolic portion of Calnexin, the ER luminal proteins were protected from proteinase‐K in the absence of TritonX‐100 in post‐digitonin pellet. Similar results were also observed in the 100,000 *g* pellet (Appendix Fig S2C and D). Moreover, if the ER membrane is damaged or ruptured due to ER stress, we could expect that it should be permeable to small metabolite such as Glutathione. As such using a version of the redox sensitive eroGFP that is attached to the ER membrane with the eroGFP facing the luminal side, we found that the redox state of eroGFP remained oxidized even during ER stress (Appendix Fig S2E). Those data indicate that during ER protein reflux, the ER membrane is not significantly damaged neither after digitonin treatment nor following differential centrifugation protocols used in our study.

**Figure 3:**
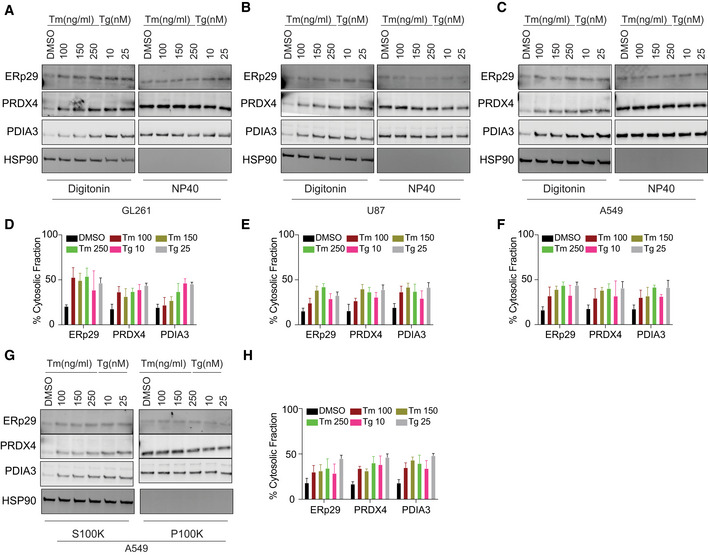
ER protein reflux is constitutive in cancer cells A–CSubcellular protein fractionation of several ER‐resident proteins in (A) GL261, (B) U87 and (C) A549 cells treated with the indicated concentrations of Tm or Tg using Digitonin. (NP40 represents the membrane fraction extracted with NP40 Cell Lysis Buffer)D–FQuantification of the subcellular protein fractionation of several ER endogenous proteins in GL261, U87, and A549 cells as in (A–C), respectively. Biological triplicates, mean ± SD calculated using Prism 9 (GraphPad).GSubcellular protein fractionation of several ER‐resident proteins in A549 cells treated with the indicated concentrations of Tm or Tg using differential centrifugation protocol.HQuantification of the subcellular protein fractionation of several ER endogenous proteins in A549 as in (G). Biological triplicates, mean ± SD calculated using Prism 9 (GraphPad).

**Figure 4:**
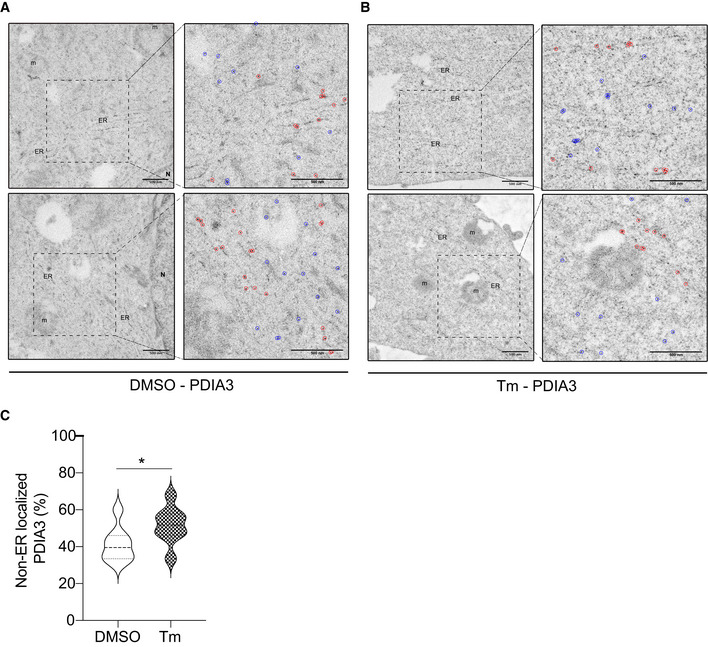
PDI proteins are redistributed to the cytosol during ER stress A, BRepresentative transmission electron microscopy images of the gold particles distribution (after immunogold labeling with PDIA3 antibodies) in A549 cells treated with DMSO or Tm. In the inserts, gold particles in the ER were surrounded by red circles and those in the rest of the cytoplasm by blue circles. (Scale bar 500 nm). n represents the nucleus, and m represents the mitochondria.CViolin plots of the gold particles distribution from the electron microscopy experiment (immunogold labeling of PDIA3) as shown in (B, C) *P‐value = 0.0486. *n* = 8 for DMSO and *n* = 8 for Tm. Thick horizontal lines represent mean ± SD ‐lighter dashed lines. Differences were analyzed by Unpaired Student’s *t*‐test using Prism 9 (GraphPad).

Next, we sought to systematically characterize the spectrum of proteins from the secretory pathway refluxed from the ER. To this end, we enriched N‐glycosylated proteins from the digitonin fraction extracted from HEK293T cells treated with Tg (Appendix Fig S2F). The purified material was subjected to mass spectrometry analysis. We focused on soluble glycoproteins that were enriched in the cytosolic fraction after Tg treatment compared with control. We identified 26 different soluble secretory N‐glycoproteins present in the cytosol (Table S2). Gene Ontology‐based analysis showed that these proteins mostly emanated from both ER and lysosomal compartments. Moreover, 23 out of these 26 proteins were part of a unique functional network (Fig 2F and Appendix Table S2), thus suggesting functional implications to this observation. We also performed a similar analysis on digitonin fractions from GBM tumor cells (isolated from patients) and compared them with our previous analysis of Tg treated HEK293T fractions. Interestingly, about 60% of hits were enriched in both fractionation approaches (Fig 2G and Appendix Table S2). This observation led us to hypothesize that during ER stress the reflux of ER proteins to the cytosol may play an important role to decrease the protein load within the ER in order to regain homeostasis. ER protein reflux was also observed in other cancer cell lines such as GL261, U87, and A549 (lung adenocarcinoma) (Fig 3A–H and Appendix Fig S3A–F) using the two aforementioned subcellular protein fractionation protocols. Next, analysis of immunogold‐labeled electron microscopy images unveiled that PDIA3 distributed to non‐ER locations in cells treated with ER stressors compared with DMSO treated cells (Fig 4A–C). It is worth to note that in cancer cells the amount of ER proteins refluxed to the cytosol was higher than in non‐cancer cells (Appendix Fig S3G).

These data indicate that upon ER stress, ER luminal proteins (and ER‐targeted sfGFP/mEOS3.2) are refluxed to the cytosol. Fluorescence microscopy with ER‐sfGFP and ER‐mEOS3.2 in HEK293T cells (Fig 2A, Appendix Fig S2A and Fig 4) confirmed the results obtained using cell fractionation and serve as alternative detergent‐free methods to monitor reflux from the ER. Moreover, the ER‐mEOS3.2 experiment showed that ER protein reflux occurred for proteins that already resided in the ER rather than as a result of the pre‐emptive quality control mechanism (Kang *et al,* 2006).

### ER stress‐mediated reflux as an ER to CYtosol Signaling (ERCYS) pathway to inhibit tumor suppressors

**Figure 5:**
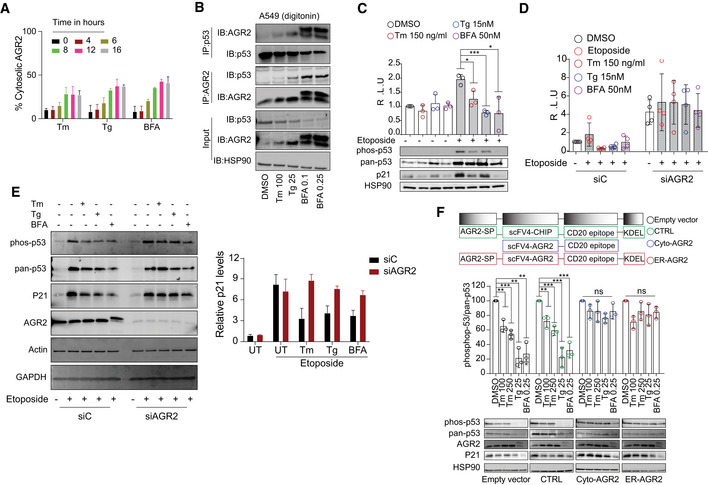
AGR2 reflux from the ER to the cytosol in A549 cells results in non‐genetic inactivation of p53 Quantification of the subcellular protein fractionation of AGR2 in A549 cells treated with Tunicamycin (Tm), Thapsigargin (Tg), and Brefeldin‐A (BFA) for different time points as shown in Appendix Fig S3A–C. *n* = 3, biological replicates (mean ± SD. Differences were analyzed using Prism 9 (GraphPad), except when otherwise indicated.Immunoprecipitation of p53 and AGR2 in the digitonin fraction of A549 cells. A549 cells were treated with Tunicamycin (Tm 100 ng/ml), Thapsigargin (Tg 25 nM), and Brefeldin A (BFA 0.1 and 0.25 nM). Endogenous p53 was immunoprecipitated from the cytoplasmic fraction (digitonin fraction) of A549 cells. Coprecipitated endogenous AGR2 was detected by Western blot.A549 were treated with Etoposide (Eto) for 2 h to induce p53 pathway. Then, cells have been challenged with Tm, Tg, and BFA at the indicated concentrations for 16 h. Luciferase experiments were performed after 24 h of transfection. Graph shows the fold induction of p53 luciferase construct. Western blot experiments for phospho‐p53, pan‐p53, p21, and HSP90 were performed as control. *n* = 3, biological replicates (mean ± SD; Differences were analyzed by Unpaired Student’s *t*‐test using Prism 9 (GraphPad), except when otherwise indicated. *P < 0.05, ***P < 0.001.A549 were transfected with control siRNA (siC) and AGR2‐tergetd siRNA (siAGR2). After 24 h, cells were transfected with p53‐luciferase construct. Cells were then treated as in (C), and luciferase experiments were performed. *n* = 4, biological replicates (mean ± SD. Differences were analyzed by Unpaired Student’s *t*‐test using Prism 9 (GraphPad), except when otherwise indicated.Left: Western blot experiments for phospho‐p53, pan‐p53, and p21 in AGR2‐silenced A549 cells exposed to ER stressors: 100 ng/ml Tunicamycin (Tm), 25 nM Thapsigargin (Tg), and 0.25 nM Brefeldin A (BFA), in the presence and absence of etoposide. Right: Quantification of the Western blot. *n* = 3, biological replicates, mean ± SD.A549 were transfected with the indicated constructs of differently targeted nanobodies, cells were then treated with Tunicamycin (Tm 100 ng/ml and 250 ng/ml), Thapsigargin (Tg 25 nM), and Brefeldin A (BFA 0.25 nM) for 16 h at the indicated concentration. Western blot experiments were performed, and HSP90 were used as loading control. *n* = 3, biological replicates mean ± SD of *n* = 3 independent experiments (***P < 0.001 and **P < 0.01). Differences were analyzed by Unpaired Student’s *t*‐test using Prism 9 (GraphPad), except when otherwise indicated.

Thus far, we have shown that ER protein reflux is constitutive in some cancer cells as is the activation of the UPR and we thus hypothesized that, as the UPR, it may play an adaptive and pro‐oncogenic function contributing to cancer cells increased fitness. To investigate possible adaptive mechanisms of the reflux process, we evaluated the nature of refluxed proteins in A549 cells subjected to ER stress (Fig 3, Appendix Fig S3A–F and Fig 5A). We focused on Anterior GRadient 2 (AGR2, PDIA17) that was highly enriched in the digitonin fractions upon ER stress (Fig 5A and Appendix Fig S3A–C). AGR2 is a PDI family member thought to catalyze protein folding through thiol‐disulfide based reactions (Chevet *et al,* 2013). In many studies, it has been shown to exert pro‐oncogenic functions through yet ill‐defined mechanisms. For instance, AGR2 was shown to interact with and to inhibit the activity of the p53 tumor suppressor (Pohler *et al,* 2004). Here, we propose a model in which ER stress in cancer cells may cause constitutive AGR2 reflux to the cytosol, where AGR2 might in turn gain new functions to interact and inhibit p53. To test this hypothesis, co‐immunoprecipitation experiments showed that upon stress AGR2 was translocated to the cytosol and interacted with wild‐type (wt) p53 in A549 cells treated with Tm, Tg, or BFA (Fig 5B). This was shown by measuring wt p53 transcriptional and p21 protein expression levels (a downstream target of p53 signaling). Tm, Tg, or BFA treatment reduced p21 protein levels, as well as wt p53 phosphorylation and transcriptional activity as shown in cells transfected with a luciferase reporter under the p53‐DNA‐binding site (Fig 5C). Moreover, AGR2‐silenced A549 cells showed increased p53 phosphorylation and p21 protein levels under ER stress conditions compared with control cells (Fig 5D and E). This confirmed that AGR2 is involved in the inhibition of wt p53 activity under ER stress. To further document that the observed inhibition of wt p53 is linked to the presence of refluxed AGR2, we engineered two nanobodies to specifically target AGR2 (either in the ER or in the cytosol) (Fig 5F). Notably, both AGR2 nanobodies showed minimal decrease in p21 protein levels and p53 phosphorylation compared to cells transfected with control nanobodies (Fig 5E). Finally, to shed the light on the physiological function of such inhibition on cancer cells fitness, we performed a sulforhodamine‐B assay in cells treated with ER stressors and etoposide for different periods of time. We found that sub‐toxic concentrations of the ER stressors decreased the toxicity caused by etoposide while in the absence of AGR2 the toxicity was increased after etoposide addition (Appendix Fig S3H and I).

These data showed that cytosolically localized AGR2 gains new functions through interacting and inhibiting wt p53 activity. As such, targeting cytosolic AGR2 in cancers could be used to restore p53 pro‐apoptotic transcriptional activity and sensitize them to existing anti‐cancer therapies. Alternatively, in pre‐neoplastic stages such as Barrett’s esophagus, in which AGR2 was first described to inhibit p53 (Pohler *et al,* 2004), the targeting of cytosolic AGR2 might prevent the inhibition of p53 tumor suppressor activity, thereby lowering cell transformation potential toward esophagus cancer.

## Discussion

The phenomenon described in this manuscript and relying on ER stress‐mediated protein reflux may act as an ER surveillance mechanism that is evolutionary conserved from yeast to mammals to achieve adaptive functions under stress conditions. This phenomenon causes ER‐resident proteins to relocate to the cytosol in different cell lines and was found to be constitutively active in cancer cell lines and in cells freshly isolated from human tumors or from murine tumor models. Moreover, the data presented herein show that this mechanism applies to a large spectrum of (glyco) proteins from the secretory pathway and it is not directly dependent/related to protein size.

**Figure 6:**
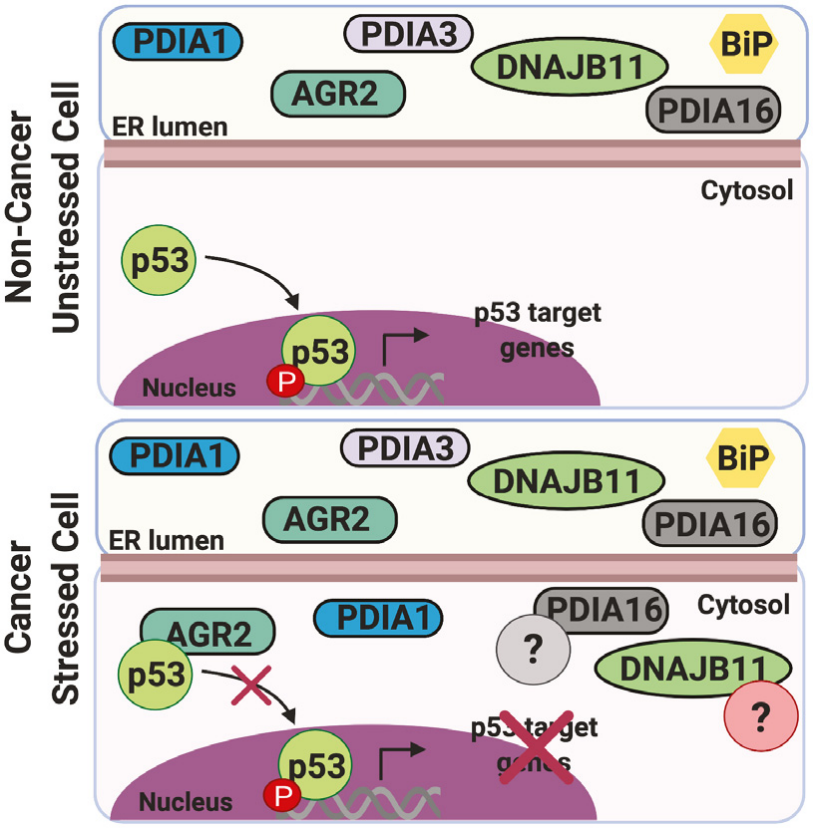
Proposed ERCYS’ working model Cartoon showing our working model, in cancer cells, basal ER stress promotes reflux of some PDI and PDI‐L proteins including AGR2 to the cytosol. In the cytosol, AGR2 is able to bind and inhibit p53 function.

ER‐to‐CYtosol‐Signaling (ERCYS) may also play an important physiological role. To date, several mechanisms were reported to decrease ER protein load either through signaling mechanisms including regulated IRE1‐dependent decay (RIDD) of RNA, PERK‐mediated global protein translation attenuation or through clearance mechanisms including pre‐emptive quality control and ERAD processes. However, here, we demonstrate that ERCYS can reduce secretory protein load in the ER lumen by refluxing folded and mature proteins to the cytosol during stress. Moreover, ERCYS‐mediated protein reflux into the cytosol is associated with selective gain‐of‐function that is pivotal for cancer development and/or progression. Indeed, this mechanism as illustrated by the cytosolic gain‐of‐function acquired by AGR2, could represent a non‐genetically mediated tumor suppression through an inhibitory interaction with p53. As such, this could be one of many other mechanisms by which refluxed ER proteins in tumor cells could influence existing pro‐apoptotic signaling pathways in the cytosol (Fig 6 and Appendix Fig S3H and I).

Beyond AGR2, another protein found to be refluxed in our study was previously shown to influence pro‐apoptotic signaling in the cytosol. Indeed, the UDP‐Glucose‐Glucosyl Transferase 1 (UGGT1) was shown to redeploy to the cytosol during enterovirus A71 (EA71) infection to help viral RNA synthesis (Huang *et al,* 2017). EA71 infection has been associated to ER stress induction (Jheng *et al,* 2018) thus supporting the hypothesis that ERCYS is an ER stress‐dependent process. In addition, one of the UGGT1 interacting proteins is the cytosolic scaffold protein DAB2IP (Hein *et al,* 2015). DAB2IP negatively regulates various signaling pathway involved in cancer progression (Bellazzo *et al,* 2017). Thus, UGGT1/DAB2IP interaction might also represent another pro‐oncogenic feature of ERCYS.

In addition, we previously reported that ER stress‐mediated protein reflux is regulated by the ER‐resident tail‐anchored HSP40 co‐chaperone Hlj1p (Igbaria *et al,* 2019). HLJ1‐like HSP40s are conserved in the human genome, and DNAJB12 (Type‐II HSP40) was characterized as an HLJ1‐like HSP40 (Grove *et al,* 2011). DNAJB12 and its homologue DNAJB14 have similar functionality as Hlj1p, and they are both required for proteasomal degradation of misfolded membrane proteins (Youker *et al,* 2004; Yamamoto *et al,* 2010; Grove *et al,* 2011). In addition, both DNAJB12 and DNAJB14 facilitate the penetration of non‐enveloped viruses from the ER to the cytosol by forming a cytosolic chaperone complex with the cytosolic heat shock protein 70, HSC70 (Goodwin *et al,* 2011; Walczak *et al,* 2014). Because ER protein reflux and the penetration of viruses from the ER to the cytosol behave similarly, we speculate that viruses hijacked an evolutionary conserved machinery—ER protein reflux—to penetrate to the cytosol.

This evidence supports the idea that ER‐resident proteins might change their localization in such specific conditions, as ER stress or virus infection, to acquire novel features associated with their new location. Further studies on the role(s) of the cytosolically localized AGR2 and other PDI‐like proteins as well as on the precise nature of the refluxed ER proteins will certainly impact on the understanding ER stress‐mediated diseases including cancer and will open new areas for biological exploration and therapeutic strategies.

## Materials and Methods

### Cell culture and transfection

Human HEK293T, A549, MCF7, U87, and mouse GL261 cell lines cells were cultured in Dulbecco’s modified Eagle’s medium (DMEM) supplemented with 10% FBS and 1% antibiotics at 37°C in a 5% CO2 incubator. PG13‐luc (WT p53 binding sites) was a gift from Bert Vogelstein (Addgene plasmid # 16442; http://n2t.net/addgene:16442; RRID: Addgene_16442). Cells were transfected with Lipofectamine LTX and plus reagent or Lipofectamine 2000 (Thermo Fisher Scientific) according to the manufacturer’s protocols. Small‐interfering RNAs (siRNA) were obtained from Ambion Each siRNA (25 nM) was transfected by reverse transfection using Lipofectamine RNAiMAX (Invitrogen). Thapsigargin (Tg), Etoposide (Eto), and Brefeldin A (BFA) were obtained from Sigma‐Aldrich (St. Louis, MO, USA). Tunicamycin (Tm) was purchased from Calbiochem.

### Immunoblot and immunoprecipitation

Whole cell extracts were prepared using RIPA buffer (25 mM Tris–HCl pH 7.5, 150 mM NaCl, 1% NP‐40, 1% sodium deoxycholate, and 0.1% SDS). Primary antibodies were incubated overnight at 4°C. Secondary antibodies were incubated 1h at RT (1:7000), and the antibodies used in this work are listed in Table 1. For the immunoprecipitation analysis, cells were lysed in Co‐IP buffer (50 mM Tris–Hcl pH 8, 150 mM NaCl, 0.5% TritonX100, and 1 mM EDTA), incubated 30’ on ice and then incubated 16 h at 4°C with the anti‐AGR2 or anti‐p53 antibodies (1 µg Ab/1,000 µg protein). After this, Dynabeads protein G/A (Life Technologies) were first washed with CoIP lysis buffer, then mixed with the protein/Ab mixture, incubated at 4°C for 3 h with gentle rotation and washed with Co‐IP buffer. Finally, the beads were eluted with 50 µl of Laemmli sample buffer, heated at 100°C for 5 min, and loaded to SDS–PAGE. For the immunoblotting, anti‐AGR2 or anti‐p53 antibodies were used.

**Table 1:** List of the antibodies used in this study

Antibody	Company
IRE1α	CST/3294S
PERK (C33E10)	CST/3192S
ATF6 (1‐7)	Abcam/ab122897
PDIA3 (Mouse)	Ptg/66423‐1‐Ig
PDIA3 (Rabbit)	Ptg/15967‐1‐AP
PDIA16	Abcam/ab134938
PDIA9	Ptg/24344‐1‐AP
PDIA1 (Mouse)	Ptg/66422‐1‐Ig
PDIA1 (Rabbit)	Ptg/11245‐1‐AP
AGR2 (Rabbit)	Ptg/12275‐1‐AP
AGR2 (Mouse)	SantaCruz/sc‐101211
DNAJB11	Ptg/15484‐1‐AP
PRDX4	Ptg/10703‐1‐AP
pan‐p53 (DO‐1) (Mouse)	SantaCruz/sc‐126
pan‐p53 (Valentino) (Rabbit)	Gift from LNCIB (Girardini *et al*, 2011)
phospho‐p53 (Ser15)	Cell signaling #9284
p21 Waf1/Cip1 (12D1)	Cell signaling #2947
Calnexin (CANX)	Gift from JJM Bergeron (McGill, Canada) (Ou *et al*, 1993)
GADPH (G‐9)	SantaCruz/sc‐365062
HSP90 (4F10)	SantaCruz/sc‐ 69703

### Tumor isolation

All human samples used for the analyses shown in this manuscript were provided by the Centre de Ressources Biologiques (CRB) Santé of Rennes (BB‐0033‐0005). Informed consent was obtained in accordance with the French legislation under the auspices of French National authorities. Mouse and human brain samples were collected and mechanically dissociated using gentleMACS dissociation following the manufacturer’s instructions (Miltenyi Biotec, Paris, France). Freshly isolated tumor tissues were suspended in 5ml DMEM immediately after being surgically removed and placed in a petri dish. Tumor and non‐tumor tissues were cut to small pieces of 1–2 mm^3^ using a sterile scalpel and then transferred to C‐tube and tightly closed. Then, we used the mechanical dissociation program A.01 for C‐tubes (the most gentle program to homogenize tissues). The resulting homogenate was then directly decanted into 40 μm cell strainer into 50ml tube. Cells were then pelleted at 100 RCF for 5 min washed and re‐pelleted for another 5 min before being subjected to 25 μg/ml digitonin as shown below in the subcellular protein fractionation protocol.

### Differential centrifugation

Cells were washed with PBS and trypsinized for 5min, pellets were collected at 300 x g and washed again with ice cold PBS. Cells were suspended in homogenizing buffer (20 mM HEPES pH7.4, 10 mM KCl, 2 mM MgCl_2_, 1 mM EDTA, 1 mM EGTA, 1 mM DTT, and protease inhibitor cocktail) and then passed 15 times through a 26‐gauge needle (1 ml syringe). After incubation for 20 min on ice, the lysates were centrifuged 300 RCF for 5 min at 4°C. The pellet (P300) contained cell debris and nuclei while the supernatant (S300) contained cytoplasm, membranes and mitochondria. S300 then was further centrifuged for 5 min at 13,000 RCF, the pellet (P13,000) contained the mitochondria and the supernatant (S13,000) contained the cytoplasm and the membrane fraction. S13,000 then was centrifuged for another 1 h at 100,000 RCF in an ultracentrifuge. After recovering the supernatant (S100K‐Cytoplasmic Fraction), the pellet (P100K‐membrane fraction) was resuspended in 400 µl of Laemmli sample buffer.

### Digitonin permeabilization

To obtain cytosolic and membrane fractions, cultured cells and dissociated cells from mice and human brains were subjected to subcellular fraction as shown in (Holden & Horton, 2009). In brief, cells were washed with cold PBS and trypsinized for 5’, collected and pellets were obtained after centrifugation at 100 RCF for 5’. Pellets were washed twice with PBS and then resuspended in Buffer 1 (see below). After 10’ in gently rotation at 4°C, tubes were subject to centrifugation at 2000 RCF at 4°C for 5’ and supernatants were harvested (Fraction 1‐ cytosol). The obtained pellets were then resuspended in Buffer 2 and incubated 30’ on ice. After 10’ centrifugation at 7500 RCF at 4°C, supernatants were harvested (Fraction 2‐membranes). Buffer 1 (cytosol)—50 mM HEPES pH 7.4, 150 mM NaCl, 10 μg/ml digitonin (add fresh). Buffer 2 (membranes)—50 mM HEPES pH 7.4, 150 mM NaCl, 1% NP40.

### Mass spectrometry analysis

*Protein digestion: After protein precipitation with acetone,* pellet was denatured with 8 M urea in 0.1 M Tris–HCl pH 8.5, reduced at 50°C with 5 mM TCEP for 30 min and alkylated with 10 mM iodoacetamide for 30 min in the dark. First digestion was performed with 5 µg endoproteinase Lys‐C (Wako) urea for 6h, followed by a 4 times dilution in Tris buffer and an overnight trypsin digestion (Promega) at a ratio 1/100. Digestion was stopped with formic acid (1% final), and peptides were desalted on Sep Pak C18 cartridge (Waters Corporation). Peptides dissolved in 0.1% TFA were quantified using colorimetric assay (Pierce – Thermo Fisher Scientific) and adjusted at 5 mg/ml. *N‐glycopeptide enrichment:* N‐glycopeptide enrichment is based on a protocol previously described (Yakkioui Y *et al*, 2017). Peptides (500 µg in 0.1 M sodium acetate pH 5.5, 150 mM NaCl) were incubated with NaIO4 for 1 h then 15 min with NaS2O3 and mixed with 100 µl AffiGel Hz Hydrazide Gel (Biorad) overnight. The unbound material was washed with tris 0.1 M pH 8.5, glycine 0.1 M, isopropanol 10% then 3 times with PBS. Lastly, PNGase F was added for 6h at 37°C and the deglycosylated peptides were extracted with 0.5% TFA. *LC‐MS analysis:* Total fraction (i.e., not Nglyco enriched) and glyco‐fraction were analyzed with an Orbitrap ELITE coupled with a nanoLC chromatographic system (Thermo Fisher Scientific). Briefly, peptides were separated on a C18 nano‐column with a linear gradient of acetonitrile and analyzed with a Top 20 CID method. Each sample was analyzed in triplicate. Data were processed by database searching against Human Uniprot Proteome database using Proteome Discoverer 2.3 software (Thermo Fisher Scientific). Precursor and fragment mass tolerance were set at 10 ppm and 0.6 Da, respectively. Trypsin with up to 2 missed cleavages was set as enzyme. Oxidation (M, +15.995 Da) and Deamidation (N, +0.984) were set as variable modification and carbamidomethylation (C, + 57.021) as fixed modification. Peptides and proteins were filtered with false discovery rate < 1%. N‐glycopeptides were filtered based on the detection of deamidation and the presence of the consensus motif NxS/T. Lastly, quantitative values obtained from extracted‐ion chromatogram (EIC) were exported in Perseus for statistical analysis.

### Immunoelectron microscopy

Cell cultures were fixed in a 2.5% PFA, 0.05% glutaraldehyde solution in 80 mM Sorensen phosphate buffer pH 7.4 for 1 h at 4°C and were scraped and washed 15 min with 0.1 M phosphate buffer. To facilitate handling of the cells, they were coated with 1.5% agarose and then cut into 1mm3 pieces. Cells were dehydrated in ethanol (30–100%) on ice and gradually infiltrated with ethanol/LR White resin (Delta microscopies) (2/1; 1/1; 1/2; successively) and finally infiltrated with pure resin overnight at 0°C. Polymerization was carried out at 60°C for 24h. Thin sections (80 nm) were collected onto 300 mesh nickel grids and processed for immunochemistry. Sections were blocked in 20mM Tris–HCl pH 7.6, 150mM NaCl (TBS buffer), containing 1% BSA, 0.1% BSA‐c™ (Aurion), 10% goat serum (Aurion), 0.2% Tween 20, twice 20 min. Grids were then incubated for 2h at room temperature with anti‐PDIA3 rabbit antibody (dilution 1:25) in Ab‐buffer (TBS pH 7.6, 1% BSA, 0.1% BSA‐c™, 1% goat serum, 0.2% Tween 20). Following four washes with the Ab‐buffer, grids were incubated for 1 h with goat antirabbit Ig conjugated to 10 nm colloidal gold (1:40 dilution) in Ab‐buffer. Sections were washed in Ab‐buffer, fixed in 2.5% glutaraldehyde and finally stained with 5% uranyl acetate and lead citrate. Negative controls were carried out, omitting primary antibodies. Sections were examined on a Tecnai Sphera operating at 200 kV (FEI, Eindhoven, Netherlands), and images were recorded with a 4x4 k CCD Ultrascan camera (Gatan, Pleasanton, USA). We used at least 8 images (x 14,500) displaying both ER and cytoplasm for each condition. The distribution of PDIA3 was estimated by calculating the ratio between the particle in the ER and the rest of the cytoplasm.

### Mouse work

Tumor cell orthotopic Implantation—Tumor cells (GL261) were implanted in the brain of immunocompetent C57BL/6rJ, 8‐week‐old male mice (Janvier, Laval, France) and tumor cells (U87) were implanted in the brain of immunodeficient mice NSG (*NOD. Cg‐Prkdcscid Il2rgtm1Wjl/SzJ*). Mice were purchased from Charles River Laboratories (Wilmington, MA), 8‐week‐old male mice (Janvier, Laval, France). All animal procedures met the European Community Directive guidelines (Agreement B35‐238‐40 Biosit Rennes, France/ No DIR 13480) and were approved by the local ethics committee and ensuring the breeding and the daily monitoring of the animals in the best conditions of well‐being according to the law and the rule of 3R (Reduce–Refine–Replace). GL261‐Luc cells were implanted in the mouse brain by intracerebral injection followed by tumor growth analysis using bioluminescence. The mice were anesthetized intraperitoneally and then fixed on a stereotactic frame. After incising the scalp, the stereotaxic coordinates were calculated for injection of tumor cells into a specific point of the brain, and reproducible for all the mice used. In the study, the tumor cells, 2,5.104 cells per mice in 1 μL for GL261‐luc and 5x104 cells per mice in 1 μL for U87, are injected at 2.2 mm to the left of the Bregma and 3.2 mm deep to perform the implantation at the level of the striatum.

### Sulforhodamine‐B (SRB) assay

This assay is adapted from (Houghton *et al*, 2007). In brief, 1X10^4^cells/well were seeded into 96‐well plate and incubated overnight. Next morning, the media were replaced by new media with different concentrations of Tm, Tg and BFA in the presence or absence of Etoposide and incubated for 24 and 48 h. At the end of each time point, cold trichloroacetic acid (TCA) acid was added to each well at a final concentration of 10% (w/v) and incubated for 1 h at 4°C. Supernatant was then discarded, and each well was washed with 100µl of distilled water for 5 times and lefted to air‐dry at room temperature. Staining was performed by adding 50 µl of 0.4% SRB (w/v) in 1% acetic acid and plates were then incubated for 30 min at room temperature. Unbound dye was washed out with 100 µl of 1% acetic acid for 5 times and air‐dried. To solubilize bound SRB dyes, 100 µl of 10mM Tris base (pH 10) was added and the plate was shaken at 500 rpm for 5 min. absorbance were measured immediately at 490nm.

### Proteinase K protection assay

Pellets obtained after the digitonin treatment (Digitonin permeabilization protocol) or after the 100,000 RCF centrifugation (Differential centrifugation protocol) were suspended well in 200μl of homogenization buffer (10 mM HEPES‐KOH (pH 7.5), 0.25 M sucrose). Proteinase‐K at a final concentration of 100 μg/ml was added to the pellets with or without 1% TritonX‐100 on ice for 30 min. At the end of the incubation time, proteins were precipitated with 10% trichloroacetic acid on ice for 30 min, and centrifuged at 12,000 RCF for 10 min at 4°C. Pellets were washed 3 times with ice cold acetone and dried. Then, pellets were resuspended in 100 μl of 3 M urea in 1X Laemmli sample buffer.

### ENDOH assay

Five volumes of proteins obtained from the digitonin fraction (cytosolic fraction) were mixed with 1 volume of 6X Laemmli buffer and boiled for 5 min. Then, sodium citrate (pH 5.5) was added to a final concentration of 67mM and samples were deglycosylated by adding 250 units of endoglycosidase‐ H (NEB) for 1 h at 37°C.

## Author contributions

Experiment design: DS, EC, and AI; Performing the experiments with FGC, RP, P‐JLR, and SC: DS; Work on murine and human tumors: RP and P‐JLR; Conducting mass spectrometry data and mass spectrometry data analysis: LN and DS; Technical support and data analysis: DT, RG, and TH; Project conceptualization, research supervision, and writing the manuscript with intellectual input and editing from all authors: EC and AI.

## Conflict of interest

EC is cofounder of Cell Stress Discoveries Ltd. (https://cellstressdiscoveries.com/).

## Supporting information



Appendix

## Data Availability

The mass spectrometry proteomics data have been deposited to the ProteomeXchange Consortium via the PRIDE partner repository with the dataset identifier PXD023567 (http://www.ebi.ac.uk/pride/archive/projects/PXD023567). The dataset is available at: http://www.ebi.ac.uk/pride.
