# Supplementary material for: Reflux of Endoplasmic Reticulum proteins to the cytosol inactivates tumor suppressors
Source: EMBO Rep. 2021 Mar 12;22(5):e51412. doi: 10.15252/embr.202051412 (PMC8724677; doi:10.15252/embr.202051412)
Supplement: Supplementary file 1 — Appendix [file EMBR-22-e51412-s001.pdf]

Appendix

Reflux of Endoplasmic Reticulum proteins to the cytosol inactivates tumor suppressors

Daria Sicari[1,2,13], Federica Grazia Centonze[1,2#] Raphael Pineau[1,2#], Pierre-Jean Le Reste[1,2,3], Luc Negroni[4,5,6,7], Sophie Chat[8], M. Aiman Mohtar[9,10], Daniel Thomas[8], Reynald Gillet[8], Ted Hupp[9,11], Eric Chevet[1,2]* and Aeid Igbaria[1,2,12]*

Appendix tables S1 – S2 2

Appendix Figure Legends S1 – S3 3

Appendix Figures S1-S3 4-6

Appendix Tables

Appendix Table S1: Characteristic of human patients involved in this study

Patient	Age at diagnosis	Gender	Symptoms	Karnofsky index	Location	IDH status	ATRX expression
1	72	Male	Aphasia	80	Left frontal lobe	WT	conserved
2	72	Male	Memory disturbance	70	Right temporal lobe	Mutated	conserved
3	65	Female	Cognitive disturbance	70	Corpus callosum	WT	conserved
4	70	Female	Seizures	100	Right frontal lobe	Mutated	conserved
5	70	Male	Cognitive disturbance	80	Left temporal lobe	WT	conserved
6	53	Male	Headache	90	Right temporal lobe	WT	conserved
7	73	Male	Cognitive disturbance	70	Right temporal lobe	WT	conserved
8	50	Male	Seizures	90	Right frontal lobe	WT	conserved
9	67	Female	Seizures, motor deficit	70	Left frontal lobe	WT	conserved

Appendix Table S2: List of the identified N-glycopeptides from the HEK293T cells treated with Tg and from the cytosolic fraction of isolated human GBM tumors.

Accession	Description	split after	baseGlyco	target	Coverage [%]	# Peptides	# PSMs	Jnique Peptic	# AAs	MW [kDa]
P14625	Endoplasmin OS=Homo sapiens OX=9606	HSP90B1	+	+	54	44	1063	43	803	92,4
Q9NYU2	UDP-glucose:glycoprotein glucosyltransferase 1 OS=Homo sapiens OX=9606	UGGT1	+	+	22	25	173	25	1555	177,1
Q96HE7	ERO1-like protein alpha OS=Homo sapiens OX=9606	ERO1A	+	+	41	15	104	14	468	54,4
Q08380	Galectin-3-binding protein OS=Homo sapiens OX=9606	LGALS3BP	+	+	25	9	78	9	585	65,3
P11047	Laminin subunit gamma-1 OS=Homo sapiens OX=9606	LAMC1	+	+	19	20	66	20	1609	177,5
Q9H3G5	Probable serine carboxypeptidase CPVL OS=Homo sapiens OX=9606	CPVL		+	27	10	60	10	476	54,1
P53634	Dipeptidyl peptidase 1 OS=Homo sapiens OX=9606	CTSC	+	+	21	6	59	6	463	51,8
P13674	Prolyl 4-hydroxylase subunit alpha-1 OS=Homo sapiens OX=9606	P4HA1	+	+	25	10	58	10	534	61
Q96AY3	Peptidyl-prolyl cis-trans isomerase FKBP10 OS=Homo sapiens OX=9606	FKBP10	+	+	24	9	47	9	582	64,2
Q8NBJ5	Procollagen galactosyltransferase 1 OS=Homo sapiens OX=9606	COLGALT1		+	15	9	43	9	622	71,6
Q9UHG3	Prenylcysteine oxidase 1 OS=Homo sapiens OX=9606	PCYOX1	+	+	17	8	41	8	505	56,6
P10253	Lysosomal alpha-glucosidase OS=Homo sapiens OX=9606	GAA		+	17	9	31	9	952	105,3
Q99538	Legumain OS=Homo sapiens OX=9606	LGMN	+	+	17	4	28	4	433	49,4
Q8NHP8	Putative phospholipase B-like 2 OS=Homo sapiens OX=9606	PLBD2	+	+	12	6	18	6	589	65,4
P08236	Beta-glucuronidase OS=Homo sapiens OX=9606	GUSB		+	8	4	12	4	651	74,7
Q12841	Follistatin-related protein 1 OS=Homo sapiens OX=9606	FSTL1		+	13	4	10	4	308	35
P07711	Cathepsin L1 OS=Homo sapiens OX=9606	CTSL		+	8	2	7	2	333	37,5
Q9H497	Torsin-3A OS=Homo sapiens OX=9606	TOR3A	+	+	7	2	5	2	397	46,2

Appendix Figure Legends

Appendix Figure S1. ER protein reflux in human and mouse derived GBM tumors. (A) schematic of the subcellular protein fractionation experiment flow. (B) Western blot for the UPR markers in total cell lysates from mice-derived tumor (T) and non-tumor tissues (NT). (C) Flow chart representation of the differential centrifugation protocol used in this study to obtain the cytosolic fraction. (D) Western blot experiments of ER-lumenal proteins of GL261-grafted-mice-derived tumor masses using differential centrifugation protocol. (E) Western blot experiments of ER-lumenal proteins of U87-grafted-mice-derived tumor masses (n=4). Tissues were isolated as described in material and methods and subjected to subcellular protein fractionation (digitonin permeabilization). Lower panel show percentage of proteins (DNAJB11, PDIA9 and PDIA3) detected in the cytosolic fraction. The horizontal line represent the sample mean. (F) Schematic of our workflow for GBM Human-patient derived tumors. (G-H) ENDOH deglycosylation assay of DNAJB11 from the cytosolic fraction isolated from murine-derived GBM tumors (G) or human derived GBM tumors (H).

Appendix Figure S2: The integrity of the ER membrane is not affected during ER stress (A)Intensity plots along the lines drawn in the confocal images in Fig.2A showing the co-localization of ER-targeted sfGFP and the cytosolically localized mCherry in these regions. Fluorescence intensity plots were generated using the software Leica LAS AF lite. (B) top: Schematic representation of the ER-targeted yemEos3.2 construct and principles of function. Bottom: representative images of cells transfected with ER targeted mEOS3.2 after a UV pulse. UV light was used to first convert the mEos3.2 then images were taken at 550 nm in the absence or presence of Tm and BFA. Scale bar 15μm (C-D) Proteinase-K protection assay on pellets obtained post digitonin fraction (C) or on the pellet after the 100K RCF centrifugation. (E) Redox state of membrane bound eroGFP on non-reducing gel vs. reducing after alkylating with N-Ethylmaleimide. (F) Proteomics experiments workflow. Right panel show Volcano plot before and after N-Glyco protein enrichment.

Appendix Figure S3: ER protein reflux in cancer cells. (A-C) Subcellular protein fractionation of several ER resident proteins in A549 cells treated with 100ng/mL Tunicamycin (Tm), 25nM Thapsigargin (Tg) or 0.25nM Brefeldin-A (BFA) for different time points using Digitonin. (D-F) Quantification of the subcellular protein fractionation of several ER endogenous proteins in A549 cells as in A-C respectively. Bars indicate the treatment in terms of hours. Biological triplicates, mean ± SD calculated using Prism 9 (GraphPad). (G) Graph show the percentage of ER-lumenal proteins in the cytosol of non -cancer (MRC5, MEF and HEK293T) and cancer cells (A549, MCF7 and GL-261) in non-stressed conditions. (H-I) Sulforhodamine-B (SRB) assay in A549 cells treated with different concentrations of Tm (100 and 250ng/mL), Tg (10, 25 and 100nM) and BFA (0.1 and 0.25nM) for 24 hours (H) or 48 hours (I) in the presence and absence of etoposide. Biological triplicates, mean ± SD calculated using Prism 9 (GraphPad).

Appendix Figures

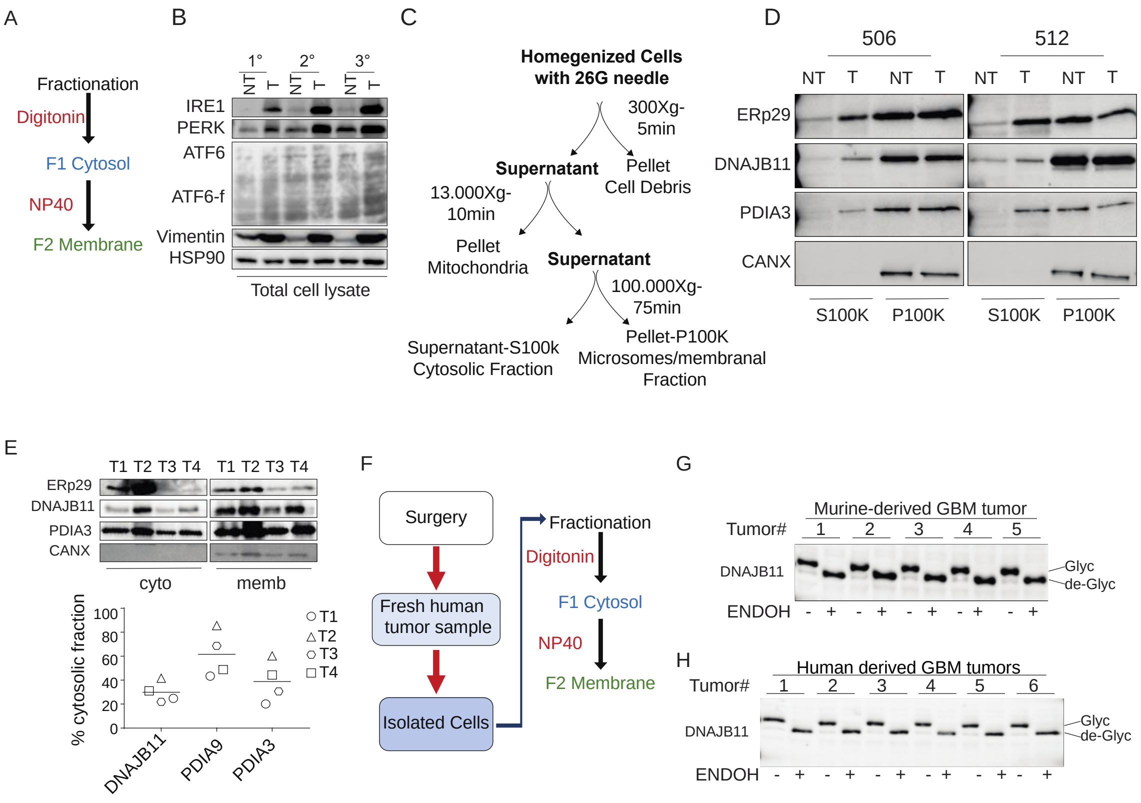

Sicari et al, Appendix Figure S1

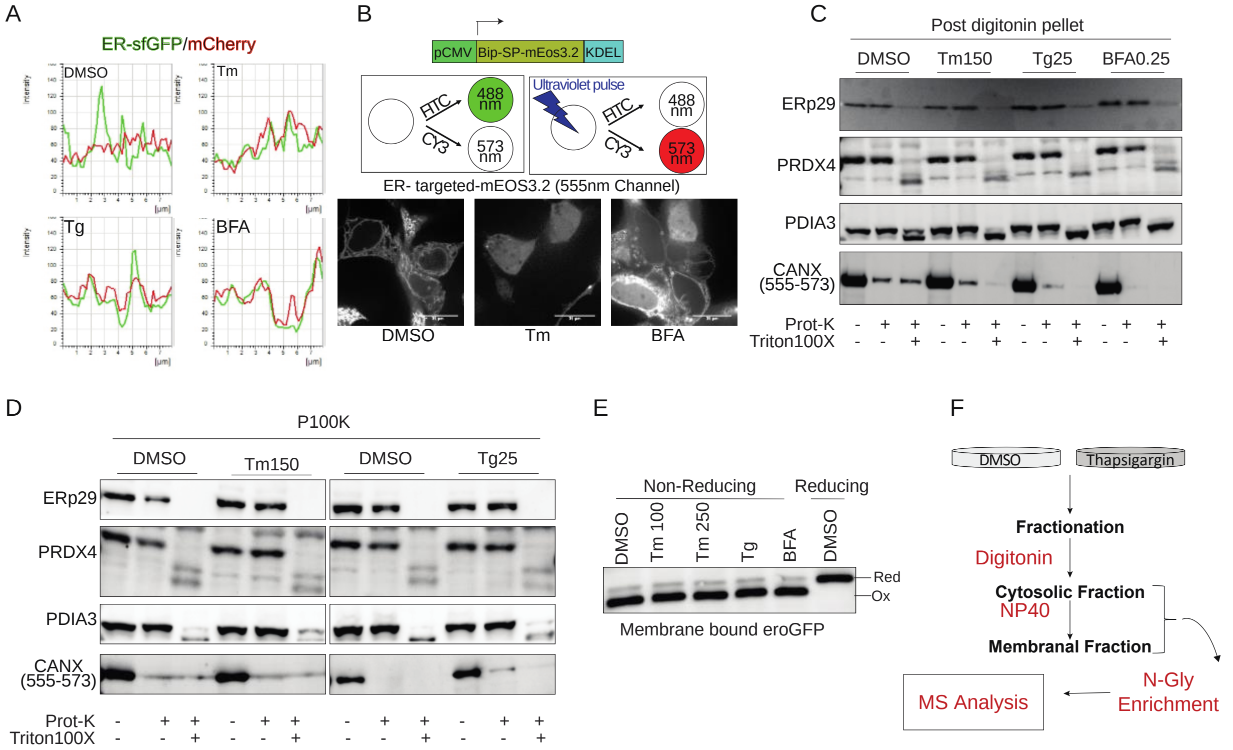

Sicari et al, Appendix Figure S2

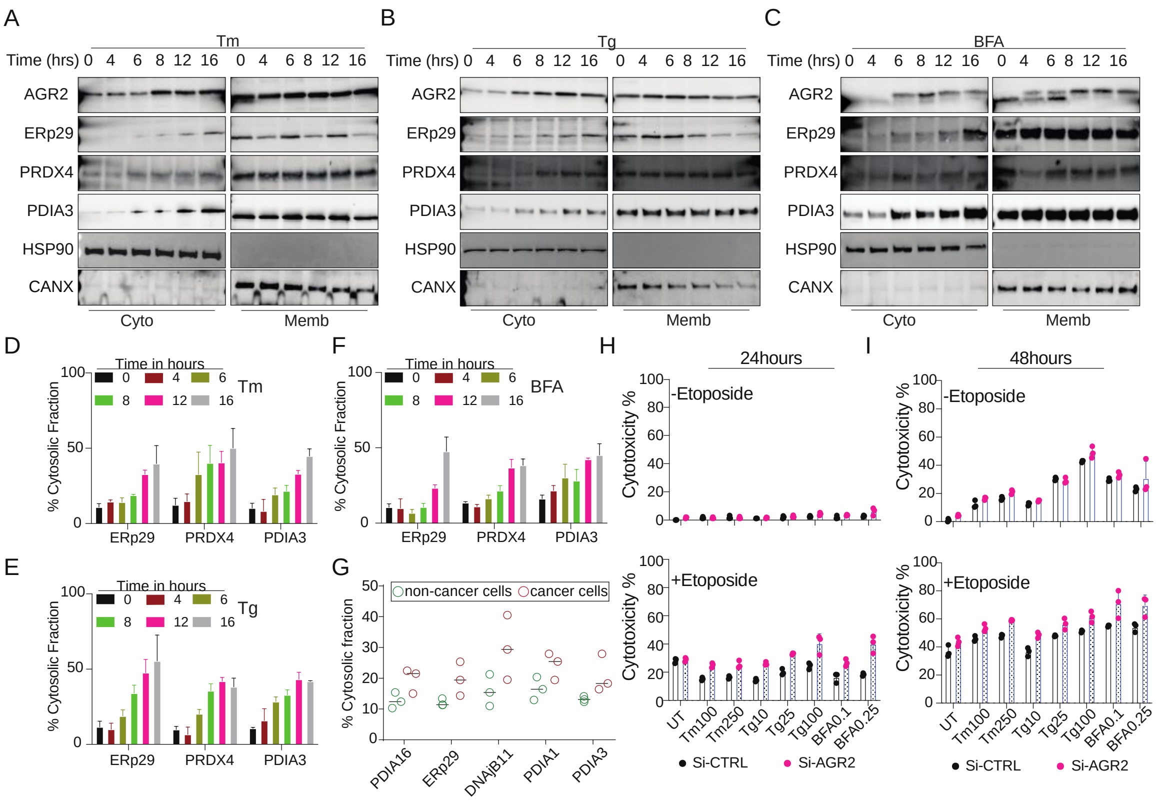

Sicari et al, Appendix Figure S3
