# Supplementary material for: Reflux of Endoplasmic Reticulum proteins to the cytosol inactivates tumor suppressors
Source: EMBO Rep. 2021 Mar 12;22(5):e51412. doi: 10.15252/embr.202051412 (PMC8724677; doi:10.15252/embr.202051412)
Supplement: Supplementary file 2 [file EMBR-22-e51412-s002.pdf]

EMBO reports

Reflux of Endoplasmic Reticulum proteins to the cytosol inactivates tumor suppressors

Daria Sicari, Federica Centonze, Raphael Pineau, Pierre-Jean Le Reste, Luc Negroni, Sophie CHAT, Aiman Mohtar, Daniel Thomas, Reynald Gillet, Ted Hupp, Eric Chevet, and Aeid Igbaria
DOI: 10.15252/embr.20 2051412

Corresponding author(s): Aeid Igbaria (aigbaria@bgu.ac.il) , Eric Chevet (eric.chevet@inserm.fr)

Review Timeline:

Transfer from Review Commons:	27th Jul 20
Editorial Decision:	7th Aug 20
Revision Received:	1st Jan 21
Editorial Decision:	4th Feb 21
Revision Received:	11th Feb 21
Accepted:	17th Feb 21

Editor: Martina Rembold

Transaction Report: This manuscript was transferred to EMBO reports following peer review at Review Commons.

(Note: With the exception of the correction of typographical or spelling errors that could be a source of ambiguity, letters and reports are not edited. Depending on transfer agreements, referee reports obtained elsewhere may or may not be included in this compilation. Referee reports are anonymous unless the Referee chooses to sign their reports.)

1st Editorial Decision **7th Aug 2020**

Dear Dr. Igbaria

Thank you for the submission of your reviewed preprint to EMBO reports. I apologize for the delay in handling your manuscript but I have now read your manuscript, the reviewer comments and your revision proposal.

Given the referees' positive assessment and the potential interest of your findings to cell and cancer biologists, I would like to invite you to submit a revised version of the manuscript, addressing all the comments of the three reviewers. We feel that it will be important to rule out that the reflux is caused by damage to the ER membrane and the experiments you described seem appropriate to address this concern. Moreover, the relevance of this pathway to cancer should be strengthened, as e.g. outlined by referee 1 in point 5 (p53-dependence of the pathway) and it should be discussed whether this is a specific and potentially regulated pathway or rather a bystander effect of unspecific reflux from the ER. In this context it might be interesting to test whether DnaJB21 and DnaJB14 play a role in reflux, although I of course note that this was not requested by the referees and it will not be required.

Please address all referee concerns in a complete point-by-point response. Acceptance of the manuscript will depend on a positive outcome of a second round of review. It is EMBO reports policy to allow a single round of revision only and acceptance or rejection of the manuscript will therefore depend on the completeness of your responses included in the next, final version of the manuscript.

Due to the delays to experimental work caused by the ongoing pandemic, we have extended our 'scooping protection policy' beyond the usual 3 month revision timeline to cover the period required for a full revision to address the essential experimental issues. This means that competing manuscripts published during the revision period will not negatively impact on our assessment of the conceptual advance presented by your study. Please contact me if you see a paper with related content published elsewhere to discuss the appropriate course of action.

IMPORTANT NOTE: we perform an initial quality control of all revised manuscripts before re-review. Your manuscript will FAIL this control and the handling will be DELAYED if the following APPLIES:

1) A data availability section is missing.
2) Your manuscript contains error bars based on n=2. Please use scatter blots showing the individual datapoints in these cases. The use of statistical tests needs to be justified.

When submitting your revised manuscript, please carefully review the instructions that follow below. Failure to include requested items will delay the evaluation of your revision.

Please note that for all articles published beginning 1 July 2020, the EMBO Reports reference style will change to the Harvard style for all article types. Details and examples are provided at https://www.embopress.org/page/journal/14693178/authorguide#referencesformat

When submitting your revised manuscript, we will require:

1) a .docx formatted version of the manuscript text (including legends for main figures, EV figures and tables). Please make sure that the changes are highlighted to be clearly visible.

2) individual production quality figure files as .eps, .tif, .jpg (one file per figure).
Please download our Figure Preparation Guidelines (figure preparation pdf) from our Author Guidelines pages
https://www.embopress.org/page/journal/14693178/authorguide for more info on how to prepare your figures.

3) a .docx formatted letter INCLUDING the reviewers' reports and your detailed point-by-point responses to their comments. As part of the EMBO Press transparent editorial process, the point-by-point response is part of the Review Process File (RPF), which will be published alongside your paper.

4) a complete author checklist, which you can download from our author guidelines (). Please insert information in the checklist that is also reflected in the manuscript. The completed author checklist will also be part of the RPF.

5) Please note that all corresponding authors are required to supply an ORCID ID for their name upon submission of a revised manuscript (). Please find instructions on how to link your ORCID ID to your account in our manuscript tracking system in our Author guidelines
()

6) Supplementary Information: please note the nomenclature "Appendix" and "Appendix Figure S1, Appendix Figure S2" etc.

Detailed instructions regarding Supplmentary information and Expanded View can be found here:

7) Please note that a Data Availability section at the end of Materials and Methods is now mandatory. In case you have no data that requires deposition in a public database, please state so instead of refereeing to the database.
See also < https://www.embopress.org/page/journal/14693178/authorguide#dataavailability>).
Please note that the Data Availability Section is restricted to new primary data that are part of this study.

8) We would also encourage you to include the source data for figure panels that show essential data. Numerical data should be provided as individual .xls or .csv files (including a tab describing the data). For blots or microscopy, uncropped images should be submitted (using a zip archive if multiple images need to be supplied for one panel). Additional information on source data and instruction on how to label the files are available .

9) Our journal encourages inclusion of *data citations in the reference list* to directly cite datasets that were re-used and obtained from public databases. Data citations in the article text are distinct from normal bibliographical citations and should directly link to the database records from which the data can be accessed. In the main text, data citations are formatted as follows: "Data ref: Smith et al, 2001" or "Data ref: NCBI Sequence Read Archive PRJNA342805, 2017". In the Reference list, data citations must be labeled with "[DATASET]". A data reference must provide the database name, accession number/identifiers and a resolvable link to the landing page from which the data can be accessed at the end of the reference. Further instructions are available at .

10) Regarding data quantification:

- Please ensure to specify the name of the statistical test used to generate error bars and P values, the number (n) of independent experiments (please specify technical or biological replicates) underlying each data point and the test used to calculate p-values in each figure legend. Discussion of statistical methodology can be reported in the materials and methods section, but figure legends should contain a basic description of n, P and the test applied.
- Graphs must include a description of the bars and the error bars (s.d., s.e.m.).
- Please also include scale bars in all microscopy images.

11) As part of the EMBO publication's Transparent Editorial Process, EMBO reports publishes online a Review Process File to accompany accepted manuscripts. This File will be published in conjunction with your paper and will include the referee reports, your point-by-point response and all pertinent correspondence relating to the manuscript.

You are able to opt out of this by letting the editorial office know (emboreports@embo.org). If you do opt out, the Review Process File link will point to the following statement: "No Review Process File is available with this article, as the authors have chosen not to make the review process public in this case."

We would also welcome the submission of cover suggestions, or motifs to be used by our Graphics Illustrator in designing a cover.

I look forward to seeing a revised version of your manuscript when it is ready. Please let me know if you have questions or comments regarding the revision.

Yours sincerely

Martina Rembold, PhD
Editor
EMBO reports

Revision 0

Review #1

1. How much time do you estimate the authors will need to complete the suggested revisions:

Estimated time to Complete Revisions (Required)

(Decision Recommendation)

Between 1 and 3 months

2. Evidence, reproducibility and clarity:

Evidence, reproducibility and clarity (Required)

The manuscript by Sicari et al shows that cancer cells exhibit a leakage (or reflux) of ER proteins to the cytosol and that this reflux is dependent or induced by the UPR. To demonstrate the pathophysiological relevance of their finding they show that one of these ER proteins, AGR2, inhibits p53. They term this phenomenon ERCYS for ER to CYtosol Signaling. The manuscript is generally clearly written and the topic is relevant and the findings are novel. I have only a few suggestions for improvements: 1- The 13,000xg pellet, which the authors discard, is likely to contain a substantial amount of ER. Thus, the authors do not have a correct estimate of the true ratio between ER and cytosol. However, this is just a small technical note, because it absolutely does not affect the major conclusion that ER proteins leak to the cytosol. 2- Figure 2: The quantification of the cytosolic fluorescence is unclear. It is evident that the reporter exhibits reflux into the cytosol, because of the signal in the nucleus. Nevertheless, it is important to report exactly how the authors have distinguished cytosolic from ER localized GFP signal. 3- The microscopy image sin Figure 4A are not of the best quality and should be replaced with better examples. 4- Why have the authors suddenly decided to use BFA in Figure 5? Its effect seems to be stronger that of Tm and Tg. A comment on this would be nice 5- Is the observed inhibition of p53 relevant for tumorigenesis? The authors are working with transformed cells, which might not be dependent on p53 inhibition. It would be very nice to show that p53 inhibition by AGR2 is an event that facilitates transformation, or that results in any other cancer hallmark like reduced apoptosis.

3. Significance:

Significance (Required)

The findings are novel and a potential pathophysiological relevance is also provided. I think that the required changes are not too many and can be done easily within a frame of 3 months. The audience taht will benefit most from the findings are primarily cell biologists, but the finding has important implications for cancer researchers. I am myself a cell biologist and found this paper of great interest to my own research.

Review #2

1. How much time do you estimate the authors will need to complete the suggested revisions:

Estimated time to Complete Revisions (Required)

(Decision Recommendation)

Between 1 and 3 months

2. Evidence, reproducibility and clarity:

Evidence, reproducibility and clarity (Required)

ER homeostasis plays important roles in cell determination. In this manuscript, the authors discovered that ER luminal proteins could be refluxed into cytosol under certain stressed or pathological conditions. The work is an extension of previous findings in yeast cells. In this case, tumor tissue and cultured mammalian cells were used. Reflux, which was subsequently termed ERCYS, was demonstrated using different methods and was proposed as an inactivation of tumor suppressors. In general, the data presented are convincing and the story is easy to follow. If proven true, ERCYS would be of great interests to a broad audience and exhibit some therapeutic potential. The manuscript could be improved if the following concerns can be addressed. **Major concerns:** 1 . The authors rely heavily on a digitonin-based protocol of cytosol extraction. Although the mechanism is plausible, it is essential to demonstrate rigorously that the digitonin-treatment is doing what it is expected to do. As the authors pointed out, changes in the composition of the ER membrane could cause different sensitization to digitonin. It would be necessary to perform immunofluorescence staining of the ER in cells that are subjected to the same digitonin treatments to see if the ER membrane retains its integrity. In addition, the authors introduced a detergent-free method using 26-gauge needle, but western blots like in Fig. 1 and 2 are needed as an essential control. 2 . Experiments shown in Fig. 2A,B requires significant improvement. In many cells, the marker exhibited a completely diffused pattern. Whether these cells are still viable is of question. In cells with a partial reflux, would it be helpful, if additional markers, such as a known cytosolic protein and an ER membrane protein, can be co-stained for comparison. Furthermore, a companion western blot of the used cells would be useful as a reference. It is also intriguing to see whether reflux would be reversed if Tm or BFA is washed off. 3 . The fact that a majority of ER-passing proteins, including secretory proteins, are identified in proteomic analysis is a bit alarming. The authors are very positive about it by saying: "Moreover, the data presented herein show that this mechanism applies to a large spectrum of (glyco)proteins from the secretory pathway. Furthermore, many of the refluxed proteins identified in our experimental systems belong to a unique functional network, suggesting functional implications to this mechanism." I would think that the same results can be interpreted as a lack of specificity. The shown variation in percentage of reflux could be easily attributed to size of the protein or differentiated affinity for membranes, etc. One simple explanation is that the reflux is caused by ER membrane rupture. Maybe one could monitor cytosolic calcium concentrations in these cells to test such possibility. In any cases, it is suggested that the authors discuss this point and tune down the conclusion. 4 . Data shown in Fig.4 did not strengthen the conclusion. It is suggested that 4A to be deleted. At the presented resolution, the colocalization can

be easily misinterpreted. It is also suggested that 4B to be moved to supplementary results. In immunogold labeling experiments, the ER membrane can not be stained, therefore making it difficult to accurately annotate the position of gold particles. 5 . In the end, the authors chose AGR2 as a refluxed example to show how ER resident protein inactivates tumor suppressors. Would expression of cytosolic AGR2 (by removing signal sequence) cause similar inhibition on p53? Why are there two bands in AGR2 blots? **Minor comments:** 1 . In Fig. S1C, the authors showed many refluxed proteins are glycosylated. It would be helpful to add enzymes to remove glycosylation in these sample and see a band shift. 2 . In Fig. 1B, it would be ideal that these blots were analyzed on the same membrane. They are currently in cropped in separate boxes. 3 . In Fig. 2, the cells were treated with various stress inducers for 24h (some says 16h in the legends). The reflux was only prominent after a long incubation. Is this always the case? 4 . The discussion is too brief.

3. Significance:

Significance (Required)

This manuscript presents an interesting phenomenon using both cultured cells and tumor tissue. As mentioned, if the reflux indeed plays a role in tumorigenesis, the findings would be of great interests. However, at the current stage, it is difficult to imagine an organized way to export folded and even glycosylated proteins across the ER membrane. It is also worrisome that the reflux could possibly be caused by membrane rupture. The observed phenotype would then be a consequence of disrupted ER homeostasis, instead of an active mechanism.

Review #3

1. How much time do you estimate the authors will need to complete the suggested revisions:

Estimated time to Complete Revisions (Required)

(Decision Recommendation)

Between 3 and 6 months

2. Evidence, reproducibility and clarity:

Evidence, reproducibility and clarity (Required)

Using a combination of biochemical and microscopy-based assays, Sicari et. al. demonstrates that ER stress causes ER-resident proteins to accumulate in the cytosol. They show that this "reflux" pathway may occur physiologically, as murine and human tumor samples, as well as cancer cell lines, all contain some level of ER-resident proteins in the cytosolic fraction. To link this phenomenon to cancer development, the authors focus on the ER-resident protein AGR2, which they suggest refluxes to the cytosol under ER stress and interacts with p53. Despite this potentially interesting observation, the major concern in this manuscript is that the overall quality of data is not strong. Conceptually, the idea that inducing ER stress (by adding Tm or Tg) can cause build-up of ER proteins in the cytosol may simply reflect damages imposed on the ER membrane that lead to

leakage - the authors need to convincingly rule out this possibility. Specific concerns that should be addressed to improve the quality of this manuscript are: 1 . In Fig. 1G, the authors present a western blot of the patient tumor samples. While the data support the presence of some ER-resident proteins in the cytosol, the lack of a non-tumor control sample makes it impossible to determine whether the protein levels are abnormal. 2 . In Fig. 2A, it is unclear how the % of cytosolic ER-sfGFP was quantified. Did the authors measure colocalization with ER and cytosol markers? If so, those channels should be shown for clarity. 3 . Several of the western blots □ such as Fig. 2C, 3A, 3B, and 3C □ qualitatively show only a slight increase in cytosolic ER proteins in Tm/Tg treated cells vs. untreated cells. Additionally, the effect of drug treatment does not increase with drug concentration. These should be clarified by the authors. 4 . In Fig. 5A, Tg treatment did not induce a significant effect on AGR2 localization to the cytosol. However, in Fig. 5B, the authors show that Tg treatment increases the interaction between p53 and AGR2. This observation lacks an explanation and suggests that Tg is exerting an off-target effect. 5 . The Hsp90 loading control in Fig. 5D is uneven and unconvincing.

3. Significance:

Significance (Required)

ER reflux is an important observation, and will have strong impact in the ER protein quality control field. However, data presented in this manuscript do not convincingly demonstrate this idea.

Inserm U1242 - Université Rennes 1
Centre de Lutte Contre le Cancer Eugène Marquis
Rue de la Bataille Flandres Dunkerque
35042 RENNES, FRANCE
Tel: +33(0)223237258
http://oss-clcc-rennes.com/

Rennes – December 15th, 2020

EMBO reports Editorial Office

Dear Dr. Rembold

We are pleased to submit a revised version of our manuscript entitled "Reflux of Endoplasmic Reticulum proteins to the cytosol yields inactivation of tumor suppressors" by Sicari et al. We provide a thorough response to the issues raised by the three reviewers, and revised the manuscript accordingly.

We feel the manuscript is much strengthened by this revision. We thank you for the handling of our manuscript and the three reviewers for their very constructive comments that helped us to improve the quality of our work. We hope that our responses and the manuscript revisions will fit the expectations of the reviewers (see point-by-point response).

Please feel free to contact us at any time if you need further information or if you have any questions.

Thank you in advance for your consideration.

Best regards,

Aeid Igbaria, Ph.D. Eric Chevet, Ph.D.

A. Igbaria

Point-by-point response to the reviewers' comments_- Preprint- #RC-2020-00244

Reviewer #1 (Evidence, reproducibility and clarity (Required)):
The manuscript by Sicari et al shows that cancer cells exhibit a leakage (or reflux) of ER proteins to the cytosol and that this reflux is dependent or induced by the UPR. To demonstrate the pathophysiological relevance of their finding they show that one of these ER proteins, AGR2, inhibits p53. They term this phenomenon ERCYS for ER to

CYtosol Signaling. The manuscript is generally clearly written and the topic is relevant and the findings are novel.
We thank this reviewer for his/her positive comments on our work.

I have only a few suggestions for improvements:

Reviewer #1 point 1: The 13,000xg pellet, which the authors discard, is likely to contain a substantial amount of ER. Thus, the authors do not have a correct estimate of the true ratio between ER and cytosol. However, this is just a small technical note, because it absolutely does not affect the major conclusion that ER proteins leak to the cytosol.
We thank the reviewer for this suggestion. We tested the 13,000xg pellet for ER proteins levels and found that the enrichment in ER proteins in those fractions were low relative to the enrichment observed in the 100,000xg pellet. In addition, since it is believed that the 13,000xg pellet would also contain mitochondria and mitochondria-associated ER and as such may present characteristics slightly different from those exhibited by the majority of the ER. In all our quantifications we measured the ratio between S100k/(P100K+S100K). Adding the P13,000xg will surely not change this ratio neither our conclusions.

Reviewer #1 point 2: Figure 2: The quantification of the cytosolic fluorescence is unclear. It is evident that the reporter exhibits reflux into the cytosol, because of the signal in the nucleus. Nevertheless, it is important to report exactly how the authors have distinguished cytosolic from ER localized GFP signal.

For the quantification, we visually scored each cell showing no-ER localization of the ER-targeted-sfGFP as being localized to the cytosol (which could be analyzed as a negative ER staining). To clarify this, we are providing here new set of experiments using the ER-targeted-sfGFP and a cytosolically localized mCherry as a marker. We visually scored any cell showing ER-sfGFP/cytosolic mCherry as being "colocalized" (yellow overlay) as was shown earlier in (Igbaria et al. 2019). This is shown now as Figure 2A and Figure S2A.

Reviewer #1 point 3: The microscopy image sin Figure 4A are not of the best quality and should be replaced with better examples.
We repeated this experiment and replaced the images with others of higher quality as shown in Figure 4A-B and Figure S2G.
Reviewer #1 point 4: Why have the authors suddenly decided to use BFA in Figure 5? Its effect seems to be stronger that of Tm and Tg. A comment on this would be nice.
All the experiments have been performed using three "canonical" ER stressors (Tm, Tg, BFA) which act through different mechanisms to ensure that our observation was not a bias introduced by the type of stressor. This was for instance illustrated by the microscopy experiments using mEOS3.2 (Figure S2A). Now we added a time course of BFA side by side with Tm and Tg as Figure S3A-C and Figure 5A. We do see that BFA treatment causes a stronger effect on protein reflux than the two other stressors. One could hypothesize that as BFA promotes the accumulation of proteins in the ER

by blocking the ER-Golgi transport, thereby enhancing the needs for debulking the ER and therefore for reflux to the cytosol.

Reviewer #1 point 5: Is the observed inhibition of p53 relevant for tumorigenesis? The authors are working with transformed cells, which might not be dependent on p53 inhibition. It would be very nice to show that p53 inhibition by AGR2 is an event that facilitates transformation, or that results in any other cancer hallmark like reduced apoptosis.

We thank the reviewer for his/her comment, it is a great suggestion. Indeed, high levels of AGR2 proteins were shown to be associated with poor prognosis of different cancers including colorectal, esophageal, lung, ovarian, breast, pancreatic, and prostate carcinomas (Pohler et al. 2004; Chevet et al. 2013; Hrstka et al. 2010). Moreover, AGR2 was reported to be associated with cell migration, cellular transformation, cell proliferation and metastasis most likely through its extracellular functions (Fessart et al. 2016). For instance, overexpression of AGR2 increases survival and proliferation of breast cancer cell lines. In the other hand, loss of function of AGR2 leads to decreased cell cycle progression and increased cell death, reviewed in (Salmans, Zhao, and Andersen 2013). As suggested by this reviewer, we performed a sulforhodamine-B assay (determination of the cellular biomass) on cells treated with ER stressors (Tm, Tg and BFA) combined with etoposide for different periods of time. We found that subtoxic concentrations of the ER stressors decreased the cytotoxicity caused by etoposide exposure while in the absence of AGR2 the toxicity was increased in the first 24hrs after etoposide addition. At the 48hrs time points we see the same notion of increased cytotoxicity in the absence of AGR2 compared to control cells. Those data are shown now as (Figure S3H-I). These results clearly indicate that AGR2 protects cells against etoposide-induced cytotoxicity during ER stress. These observations along with our results presented in Figure 5 make AGR2 an important player during ER to facilitate tumorigenesis.

Reviewer #1 (Significance (Required)):

The findings are novel and a potential pathophysiological relevance is also provided. I think that the required changes are not too many and can be done easily within a frame of 3 months. The audience taht will benefit most from the findings are primarily cell biologists, but the finding has important implications for cancer researchers. I am myself a cell biologist and found this paper of great interest to my own research.

Again, we deeply thank this reviewer for his/her constructive and positive comments on our work.

==

Reviewer #2 (Evidence, reproducibility and clarity (Required)):

ER homeostasis plays important roles in cell determination. In this manuscript, the authors discovered that ER luminal proteins could be refluxed into cytosol under certain stressed or pathological conditions. The work is an extension of previous findings in yeast cells. In this case, tumor tissue and cultured mammalian cells were used. Reflux, which was subsequently termed ERCYS, was demonstrated using different methods and was proposed as an inactivation of tumor suppressors. In

general, the data presented are convincing and the story is easy to follow. If proven true, ERCYS would be of great interests to a broad audience and exhibit some therapeutic potential. The manuscript could be improved if the following concerns can be addressed.
We thank this reviewer for his/her positive comments on our work.

Major concerns: ** **Reviewer #2 point 1: The authors rely heavily on a digitonin-based protocol of cytosol extraction. Although the mechanism is plausible, it is essential to demonstrate rigorously that the digitonin-treatment is doing what it is expected to do. As the authors pointed out, changes in the composition of the ER membrane could cause different sensitization to digitonin. It would be necessary to perform immunofluorescence staining of the ER in cells that are subjected to the same digitonin treatments to see if the ER membrane retains its integrity. In addition, the authors introduced a detergent-free method using 26-gauge needle, but western blots like in Fig. 1 and 2 are needed as an essential control.
We totally understand the reviewer's concerns for using digitonin and this was also one of our concerns during the realization of this work. As such it was important for us too to make sure that digitonin is doing what it is expected to do and did not alter significantly the permeability of the ER membrane which would introduce a major flaw in our conclusions. To this end, we performed several control experiments of which some were included in the previous version of the manuscript and some other are now presented in the revised version. No matter the protocol used, the results obtained were similar regarding the presence of ER resident proteins in the cytosol as shown in revised Figure 2B & 2D for digitonin compared to Figure 2C & 2E for differential centrifugation. An additional confirmation is presented in Figure 3C & 3F for digitonin compared to Figure 3G-H for differential centrifugation.

Regarding subcellular fractionation in tumors, we had to choose one method to perform the experiments in order to accommodate the facts that those samples are both in low quantity and of limited accessibility (especially for GBM patient tumors). As such we preferentially used the digitonin method that is faster and more effective (yields of proteins recovered) than the differential centrifugation method. The efficacy issue was also very important to process many samples in parallel. Indeed, in the case of the mouse tumors we were dealing with many samples at a time considering that each control (non-tumor) should be processed at the same time as the tumors, which complicated the logistics of the sample processing. To minimize sample processing time as much as possible between the time the tumors were extracted and the production of the cytosolic and membrane fractions.
In the revised version of the manuscript we have added the following experiments:

1. control experiments were performed in two different murine GBM tumors using the differential centrifugation protocol as shown in (Figure S1C-D).
2. a proteinase-K protection assay was carried out as we reasoned that if the ER membrane is damaged/ruptured due to digitonin or differential centrifugation protocols this will cause ER lumenal proteins to be sensitive for proteinase-K-mediated proteolysis. As shown in (Figure S2C-D), proteinase K was active towards the cytosolic domain of Calnexin, while the ER lumenal proteins were protected against proteinase-K in the absence of TritonX100 in both post-digitonin and 100,000xg pellets. These data demonstrate that the ER

membrane is intact and is not damaged after digitonin or differential centrifugation protocols.

Reviewer #2 point 2: Experiments shown in Fig. 2A,B requires significant improvement. In many cells, the marker exhibited a completely diffused pattern. Whether these cells are still viable is of question. In cells with a partial reflux, would it be helpful, if additional markers, such as a known cytosolic protein and an ER membrane protein, can be co-stained for comparison. Furthermore, a companion western blot of the used cells would be useful as a reference. It is also intriguing to see whether reflux would be reversed if Tm or BFA is washed off.

We repeated those experiments with additional controls, please refer to our response to **Reviewer #1 point 2 above**. Unfortunately, we will not be able to add a cytosolic marker for the images used in Figure 2B as we are using mEOS3.2 that has excitation maxima at 488nm, we use the 405nm wavelength to photoconvert it to the 570nm channel which make it difficult to add any other channel. We are convinced that those cells are still viable for several reasons including the facts that they are not rounded up and they still adherent to the bottom of the dish. In addition, as shown in the revised Figure S3H-I and explained in our response to Reviewer #1 point 5, at 24hrs post treatment with ER stressors (Tm, Tg and BFA) at subtoxic concentrations, we observed less than 5% of cell toxicity as measured using sulforhodamine-B staining. We must emphasize that our microscopy imaging was done 24hrs post treatment with ER stressors (Tm, Tg and BFA) hence we expect a cytotoxicity similar/identical to that observed at the 24hrs time point. Regarding the wash out experiment, we believe this is a valid question that should be addressed. We treated cells for 16 hrs, wash out the ER stressors and then monitor the localization of several ER lumenal proteins at 24 and 48 hours post wash out. We found that when the stressor are washed out the amount of the refluxed proteins to the cytosol decreased slightly after 24hours. Waiting 48hrs after wash had almost depleted the cytosol of the tested ER proteins as shown in Figure-REVIEWERS ONLY-B. We do not believe that the cytosolically refluxed protein would go back to the ER as they lack all known signal peptides that can target them to the ER. Following the fate of the refluxed proteins is our high interest for future exploration.

Reviewer #2 point 3:. The fact that a majority of ER-passing proteins, including secretory proteins, are identified in proteomic analysis is a bit alarming. The authors are very positive about it by saying: "Moreover, the data presented herein show that this mechanism applies to a large spectrum of (glyco)proteins from the secretory pathway. Furthermore, many of the refluxed proteins identified in our experimental systems belong to a unique functional network, suggesting functional implications to this mechanism." I would think that the same results can be interpreted as a lack of specificity. The shown variation in percentage of reflux could be easily attributed to size of the protein or differentiated affinity for membranes, etc. One simple explanation is that the reflux is caused by ER membrane rupture. Maybe one could monitor cytosolic calcium concentrations in these cells to test such possibility. In any cases, it is suggested that the authors discuss this point and tune down the conclusion.

We thank Reviewers #2 for his/her input on this, and we edited the discussion to add more clarifications regarding these issues. As we use chemicals that change the

concentrations of calcium in the cytosol such as Tg, we found that it is better we follow redox changes inside ER lumen rather than calcium concentrations in the cytosol to rule out the possibility that this mechanism is caused by a damaged ER or ruptures the ER membrane. For this, we used similar system to what we used earlier in yeast (Igbaria et al. 2019) relying on a version of redox sensitive eroGFP that is attached to the ER membrane reasoning that if the ER membrane becomes damaged and leaky for small metabolites like the cytosolic glutathione then this will cause a change in the redox environment within the ER and reduce the sensitive cysteines within the eroGFP. As shown in the revised Figure S2E we did not observe any change in the redox status of eroGFP under ER stress conditions, thereby confirming the integrity of the membrane.

Reviewer #2 point 4: Data shown in Fig.4 did not strengthen the conclusion. It is suggested that 4A to be deleted. At the presented resolution, the colocalization can be easily misinterpreted. It is also suggested that 4B to be moved to supplementary results. In immunogold labeling experiments, the ER membrane cannot be stained, therefore making it difficult to accurately annotate the position of gold particles.
Please refer to our response to **Reviewer #1 point 3.** We repeated this experiment and replaced the images with others of higher quality as shown in Figure 4A-B.

Reviewer #2 point 5: In the end, the authors chose AGR2 as a refluxed example to show how ER resident protein inactivates tumor suppressors. Would expression of cytosolic AGR2 (by removing signal sequence) cause similar inhibition on p53? Why are there two bands in AGR2 blots?
As shown in the revised Figure 5F, targeting cytosolic AGR2 with specific nanobodies against AGR2 in the cytosol (without affecting the ER-lumenal AGR2 protein) was sufficient to restore the activity of p53. Although overexpression of AGR2 alone may not be capable of inhibiting p53 (as we do not know yet what targets AGR2 reflux, its binding to p53 and whether other ER proteins and cytosolic adaptors are needed for this), we overexpressed cytosolically-located AGR2 (devoid of signal peptide) and measure p53 phosphorylation and p21 protein levels in the presence and absence of etoposide. Although we do see that the cytosolic AGR2 is highly expressed we do not see any decrease in wt-p53 phosphorylation or decrease in p21 protein levels as expected and as seen in the presence of ER stress. One explanation could be that AGR2 might have to enter the ER to reach a conformation which, once the protein localized in the cytosol, will allow its binding to p53. Actually these finding strength our conclusion that the AGR2 that is active in the cytosol have to originate from the ER. This experiment is provided here as “FIGURE FOR REVIEWERS ONLY-A”.
Regarding the two immune-reactive bands observed for AGR2 upon treatment with BFA, this is still a mystery for us. We believe that AGR2 could acquire specific post-translational modifications under those conditions as previously shown for instance with O-glycosylation (Clarke, Rudland, and Barraclough 2015), however this would at this stage need further work to provide robust and significant information.

****Minor comments:****

Reviewer #2 point 1 (minor comments). In Fig. S1C, the authors showed many refluxed proteins are glycosylated. It would be helpful to add enzymes to remove glycosylation in these sample and see a band shift.
We performed ENDOH treatment on the cytosolic fractions obtained from the murine GL261 derived GBM tumors and from the cytosolic fraction obtained from human GBM tumors. We found that in all these samples, DNAJB11 glycosylation in the cytosolic fraction was removed upon exposure to ENDOH thereby indicating that those proteins are present in their glycosylated form in the cytosol. Those data were added in the revised version of our manuscript in Figure S1G-H.

Reviewer #2 point 2 (minor comments). In Fig. 1B, it would be ideal that these blots were analyzed on the same membrane. They are currently in cropped in separate boxes.
Lysates were analyzed on the same membrane and the new results are presented in the revised version of the manuscript as Figure 1B.

Reviewer #2 point 3 (minor comments). In Fig. 2, the cells were treated with various stress inducers for 24h (some says 16h in the legends). The reflux was only prominent after a long incubation. Is this always the case?
We have added information about the kinetics of protein reflux in the revised Figure S3A-C and Figure 5A.

Reviewer #2 point 3 (minor comments). The discussion is too brief.
The discussion was written in a report format, we edited the discussion and respect the word count limits.

Reviewer #2 (Significance (Required)):
This manuscript presents an interesting phenomenon using both cultured cells and tumor tissue. As mentioned, if the reflux indeed plays a role in tumorigenesis, the findings would be of great interests. However, at the current stage, it is difficult to imagine an organized way to export folded and even glycosylated proteins across the ER membrane. It is also worrisome that the reflux could possibly be caused by membrane rupture. The observed phenotype would then be a consequence of disrupted ER homeostasis, instead of an active mechanism.
We thank Reviewer #2 for his/her acknowledgement of how significant is our reported phenomenon. We would like to emphasize few points that may help to waive some of this reviewer's concerns about the export of large glycoproteins through the ER membrane. We previously discussed this point in our manuscript (Igbaria et al. 2019). In yeast we found that the reflux process is regulated by the HSP40 protein named HLJ1, the putative functional homologs of HLJ1 in humans are DnaJB12 and DnaJB14. Both DNAJB proteins were shown to mediate the penetration of non-enveloped viruses from the ER to the cytosol (Walczak et al. 2014). Due to the similarity of the ER protein reflux and the penetration of viruses from the ER to the cytosol, we speculate that ER protein reflux machinery may be seized/hijacked by some viruses to penetrate to the cytosol. This is important because it was also reported that during the process of nonenveloped viruses penetration, large and intact viral particles are able to penetrate the ER membrane on their way to the cytosol (Inoue and Tsai 2011) such as the VP1

protein. Moreover, we added more clarification in the discussion part of the revised manuscript and cited the literature on another large glycoproteins like the UGGT1 (171 kda) that is known to deploy to the cytosol upon viral infection(Huang et al. 2017). We must note that UGGT1 was independently found in our mass spectrometry analysis of the digitonin fraction obtained from HEK293T cells treated with Tg and from isolated human GBM tumors.

===

Reviewer #3 (Evidence, reproducibility and clarity (Required)):

Using a combination of biochemical and microscopy-based assays, Sicari et. al. demonstrates that ER stress causes ER-resident proteins to accumulate in the cytosol. They show that this "reflux" pathway may occur physiologically, as murine and human tumor samples, as well as cancer cell lines, all contain some level of ER-resident proteins in the cytosolic fraction. To link this phenomenon to cancer development, the authors focus on the ER-resident protein AGR2, which they suggest refluxes to the cytosol under ER stress and interacts with p53. Despite this potentially interesting observation, the major concern in this manuscript is that the overall quality of data is not strong. Conceptually, the idea that inducing ER stress (by adding Tm or Tg) can cause build-up of ER proteins in the cytosol may simply reflect damages imposed on the ER membrane that lead to leakage - the authors need to convincingly rule out this possibility.

We thank this reviewer for his/her comments. We totally understand his/her concerns and we addressed most of the raised issues in the text below as well as in our responses to Reviewer #1 and Reviewer #2.

Specific concerns that should be addressed to improve the quality of this manuscript are:

Reviewer #3 point 1. In Fig. 1G, the authors present a western blot of the patient tumor samples. While the data support the presence of some ER-resident proteins in the cytosol, the lack of a non-tumor control sample makes it impossible to determine whether the protein levels are abnormal.

Unfortunately, we do not have access to fresh non-tumor tissues from human patients as the removal of “normal” brain tissue is very rarely performed. Alternatively, we did have access to those controls in the murine tumor model by taking the healthy hemisphere of the brain as non-tumor control. In addition, as shown in the human tumors data, we found that high percentages (sometimes more than 50%) of ER lumenal proteins that are known to exert specific activities inside the ER lumen were found in the cytosol. This observation by itself is very interesting and opens a lot of areas to explore regarding their new activities in the cytosol. Those proteins usually reside in the ER lumen to carry several functions during oxidative protein folding and their function in the cytosol is poorly studied. In our manuscript, we demonstrated such gain of cytosolic function of an ER protein in the cytosol, and the results are presented in the revised Figure 5. We show that AGR2, an ER targeted member of the PDI family, acts as an inhibitor of p53 transcriptional activity.

Reviewer #3 point 2. In Fig. 2A, it is unclear how the % of cytosolic ER-sfGFP was

quantified. Did the authors measure colocalization with ER and cytosol markers? If so, those channels should be shown for clarity.

Please refer to our response to Reviewer #1 point 2. For the quantification, we visually scored each cell showing no ER localization of the ER-targeted-sfGFP as corresponding to a localization to the cytosol (which could be analyzed as a negative ER staining). To clarify this, we are providing here new set of experiments using the ER-targeted-sfGFP and a cytosolically localized mCherry as a marker. We visually scored any cell showing ER-sfGFP/cytosolic mCherry as being "colocalized" (yellow overlay) as was shown earlier in (Igbaria et al. 2019). This is shown now as Figure 2A and Figure S2A.

Reviewer #3 point 3. Several of the western blots – such as Fig. 2C, 3A, 3B, and 3C – qualitatively show only a slight increase in cytosolic ER proteins in Tm/Tg treated cells vs. untreated cells. Additionally, the effect of drug treatment does not increase with drug concentration. These should be clarified by the authors.

Regarding Figures 2C, 3A, 3B, and 3C, please refer to the quantifications provided (Figures 2E, 3D, 3E and 3F, respectively). Some of the proteins like ERp29 are not expressed at high levels in several cell lines and qualitatively this leads to the impression that the cytosolic increase it is not important. To this end, it is best to take into consideration the quantifications. Moreover, as we demonstrated before in our yeast work (Igbaria et al. 2019), we do believe that reflux is a saturable mechanisms. We showed that beyond a threshold (that remains to be defined) the increase of ER stress does not increase the amount of protein refluxed. Finally, we observe this phenomenon with the reflux kinetics measured for some proteins (for PRDX4 with Tm, and BFA and for AGR2) where longer exposure to ER stress does not increase the cytosolic accumulation of these proteins (Figure S3A-F and Figure 5A).

Reviewer #3 point 3. In Fig. 5A, Tg treatment did not induce a significant effect on AGR2 localization to the cytosol. However, in Fig. 5B, the authors show that Tg treatment increases the interaction between p53 and AGR2. This observation lacks an explanation and suggests that Tg is exerting an off-target effect.

We thank this reviewer for pointing this out. As shown in the revised Figure 5B, in the input lane (which corresponds to the cytosolic fraction) we do see that AGR2 is highly refluxed to the cytosol with the different ER stressors used including Tm, Tg and BFA. This information was missing in the previous Figure 5A and now we provide a full kinetics of AGR2 reflux in A549 cells upon treatment with Tm, Tg or BFA at different time points as shown in the revised Figure S3A-C and Figure 5A.

Reviewer #3 point 3. *The Hsp90 loading control in Fig. 5D is uneven and unconvincing*.

HSP90 loading control will be replaced by GAPDH control in the revised Figure 5E.

Reviewer #3 (Significance (Required)):

ER reflux is an important observation, and will have strong impact in the ER protein quality control field. However, data presented in this manuscript do not convincingly demonstrate this idea.

Again, we thank reviewer #3 for the positive and constructive comments and his/her acknowledgment of how significant is the data we are reporting in our manuscript. We do hope that we addressed all of his/her concerns.

References:

Chevet, E., D. Fessart, F. Delom, A. Mulot, B. Vojtesek, R. Hrstka, E. Murray, T. Gray, and T. Hupp. 2013. 'Emerging roles for the pro-oncogenic anterior gradient-2 in cancer development', *Oncogene*, 32: 2499-509.

Clarke, C., P. Rudland, and R. Barraclough. 2015. 'The metastasis-inducing protein AGR2 is O-glycosylated upon secretion from mammary epithelial cells', *Mol Cell Biochem*, 408: 245-52.

Fessart, D., C. Domblides, T. Avril, L. A. Eriksson, H. Begueret, R. Pineau, C. Malrieux, N. Dugot-Senant, C. Lucchesi, E. Chevet, and F. Delom. 2016. 'Secretion of protein disulphide isomerase AGR2 confers tumorigenic properties', *Elife*, 5.

Hrstka, R., R. Nenutil, A. Fourtouna, M. M. Maslon, C. Naughton, S. Langdon, E. Murray, A. Larionov, K. Petrakova, P. Muller, M. J. Dixon, T. R. Hupp, and B. Vojtesek. 2010. 'The pro-metastatic protein anterior gradient-2 predicts poor prognosis in tamoxifen-treated breast cancers', *Oncogene*, 29: 4838-47.

Huang, P. N., J. R. Jheng, J. J. Arnold, J. R. Wang, C. E. Cameron, and S. R. Shih. 2017. 'UGGT1 enhances enterovirus 71 pathogenicity by promoting viral RNA synthesis and viral replication', *PLoS Pathog*, 13: e1006375.

Igbaria, A., P. I. Merksamer, A. Trusina, F. Tilahun, J. R. Johnson, O. Brandman, N. J. Krogan, J. S. Weissman, and F. R. Papa. 2019. 'Chaperone-mediated reflux of secretory proteins to the cytosol during endoplasmic reticulum stress', *Proc Natl Acad Sci U S A*, 116: 11291-98.

Inoue, T., and B. Tsai. 2011. 'A large and intact viral particle penetrates the endoplasmic reticulum membrane to reach the cytosol', *PLoS Pathog*, 7: e1002037.

Pohler, E., A. L. Craig, J. Cotton, L. Lawrie, J. F. Dillon, P. Ross, N. Kernohan, and T. R. Hupp. 2004. 'The Barrett's antigen anterior gradient-2 silences the p53 transcriptional response to DNA damage', *Mol Cell Proteomics*, 3: 534-47.

Salmans, M. L., F. Zhao, and B. Andersen. 2013. 'The estrogen-regulated anterior gradient 2 (AGR2) protein in breast cancer: a potential drug target and biomarker', *Breast Cancer Res*, 15: 204.

Walczak, C. P., M. S. Ravindran, T. Inoue, and B. Tsai. 2014. 'A cytosolic chaperone complexes with dynamic membrane J-proteins and mobilizes a nonenveloped virus out of the endoplasmic reticulum', *PLoS Pathog*, 10: e1004007.

Figure for reviewers only

A

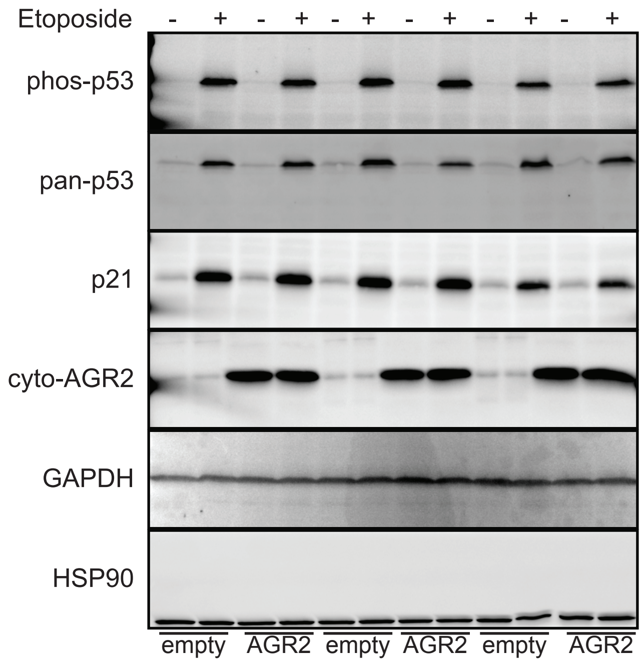

B

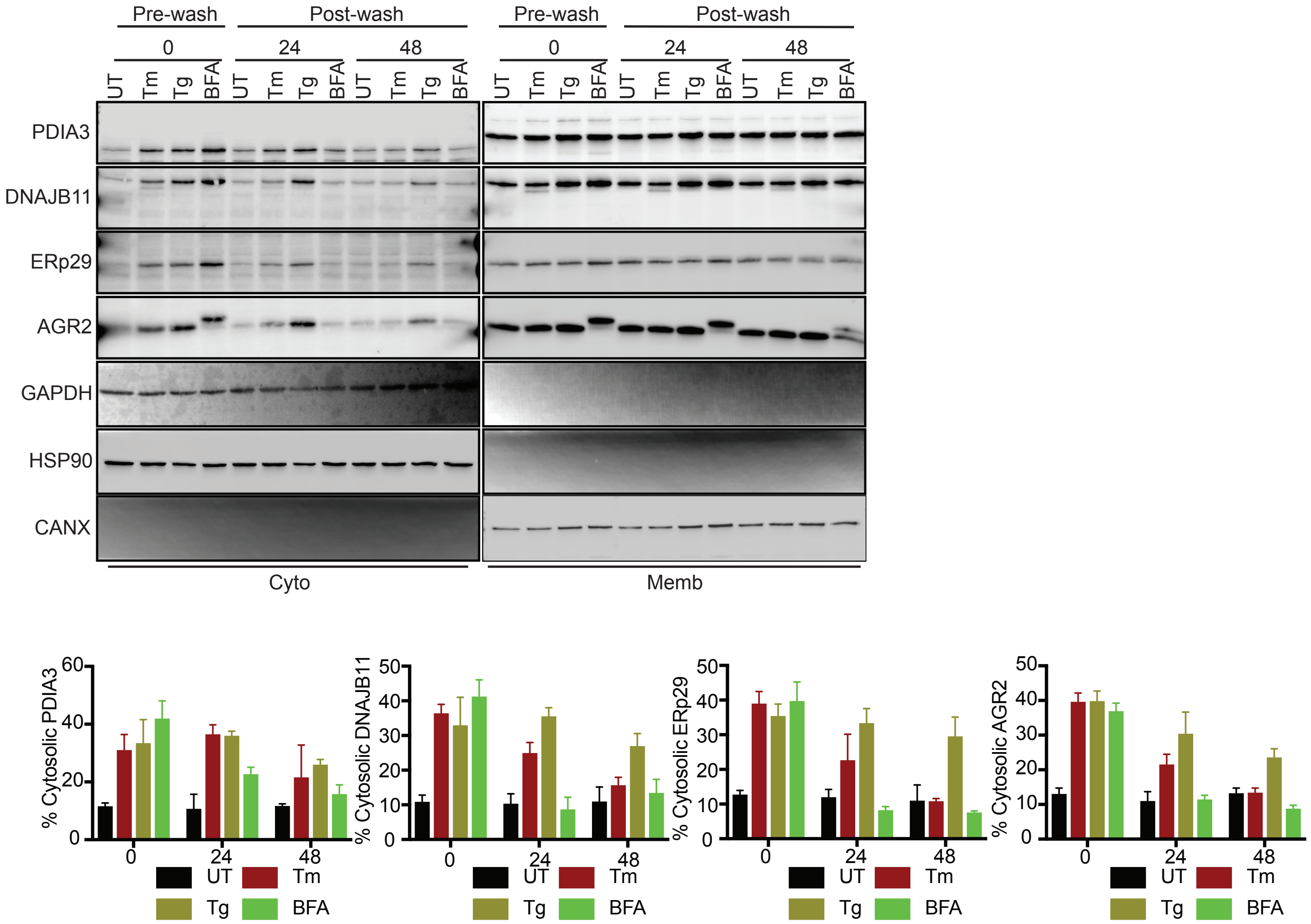

1st Revision - Editorial Decision 4th Feb 2021

Dear Dr. Igbaria

Thank you for the submission of your revised manuscript to EMBO reports. We have now received the full set of referee reports that is copied below.

As you will see, all referees are now positive about the study and request only minor changes to clarify text and figures. If you decide to keep Fig. 4A,B, please justify this in a point-by-point response and please be cautious when describing these data.

From the editorial side, there are also a few things that we need before we can proceed with the official acceptance of your study:

1) Your article currently has 5 figures and will therefore be published in our Reports section, therefore please combine the Results and Discussion section. If you feel that an extended discussion section is essential, you can also resubmit the manuscript with 6 figures (e.g., a separate figure for the model). In this case you can leave the Results and Discussion section separate.

2) Please provide up to five keywords

3) Please provide Conflict of Interest and Author Contribution paragraphs.

4) Please move the Figure legends to the end of the manuscript (after the references)

5) Please update the reference format to match the style of EMBO Reports (list the first 10 authors followed by et. al. and put the year in brackets).
See also

6) Please update the relevant fields on funding information in our online submission system.

7) Supplementary Information:

- Please change the nomenclature to "Appendix" and "Appendix Figure Sx", "Appendix Table Sx". Please also update the relevant callouts in the manuscript text.

- Please add page numbers to the table of content on the first page.

- Please move the Supplementary materials and methods and the antibody table to the Material and Methods section of the main manuscript.

- Legends:
S1B: please define 'NT' and 'T'
S1E: please define the number and nature of the experiments and the horizontal black line
S2B: please provide a scale bar
S3A: please define the concentrations for Tm, Tg, and BFA
S3D-F: please define the bars and error bars and the number of experiments
S3H, I: please define the units for the concentration of Tm etc (100 xx), the number of experiments and the nature of bars and error bars.

8) Author checklist/ethics statements:

- The sentence in 4.a appears incomplete ("For all experiments requiring")

- Please complete section D - Animal models in the checklist since you report on tumor cell implantation experiments in mice.

- Please add an ethics and approval statement regarding the human tumor samples in the Author checklist and in the methods section of the manuscript.

9) Data availability section: Please add a link that resolves to the dataset.

10) I attach a document with some comments and edits in the figure legends. You have already replied to most of the comments from our data editors before the manuscript was re-reviewed but some points remain unresolved and I also added a few more items you need to address. Moreover, I took the liberty to suggest some changes to the title and abstract. Please review these.

11) Finally, EMBO reports papers are accompanied online by A) a short (1-2 sentences) summary of the findings and their significance, B) 2-3 bullet points highlighting key results and C) a synopsis image that is 550x200-600 pixels large (width x height) in .png format. You can either show a model or key data in the synopsis image. Please note that the size is rather small and that text needs to be readable at the final size. Please send us this information along with the revised manuscript.

We look forward to seeing a final version of your manuscript as soon as possible.

Your sincerely,

Martina Rembold, PhD
Senior Editor
EMBO reports

****

Referee #1:

I went through the point-by-point response to my original comments and through the revised manuscript. As far as I can see, the authors have addressed all my initial comments and I have no further comments or suggestions.

Referee #2:

The authors have performed additional experiments to address most of my concerns. In particular, they added proteinase K experiments and redox-sensitive probes, trying to strengthen the argument that the observed phenomenon is not due to membrane rupture/leakage. In general, I find these efforts acceptable. There are still a few minor comments for the authors to consider and revise:

1) When calnexin was digested by proteinase K, a lower band would often appear, if the authors saw this band, it would be helpful to include it in the blot. The disappearance of the full-length band should correlate with the accumulation of the lower band.
2) Many text labels in the figures are too close to lines or blots. For exmaple, in Fig 1B, the "NT" and "T" are stepping on the blot below. please adjust accordingly.
3) The label in S2E is misplaced, please fix.
4) I still find Fig. 4A,B problematic. The relocation of the PDIA3 is very difficult to see. The Pearson analysis used rely heavily on the levels of these fluorescent signals. For example, ER inducer suppose to increase the levels of calnexin and PDI, but they are induced to different extents, it could easily influence the coefficients. Furthermore, there is an apparent nuclear import of PI3K upon stress. The reduction of the coefficients could be due to the reduction of cytosolic PI3K, instead of the increase of cytosolic PDIA3. Please consider deleting this part as previously suggested.

Referee #3:

This revision has largely addressed most of my previous concerns.

Point-by-point response to the Editor’s and reviewers’ comments_- Preprint-#RC-2020-00244

Editor’s comments:
Thank you for the submission of your revised manuscript to EMBO reports. We have now received the full set of referee reports that is copied below.
As you will see, all referees are now positive about the study and request only minor changes to clarify text and figures. If you decide to keep Fig. 4A,B, please justify this in a point-by-point response and please be cautious when describing these data.
From the editorial side, there are also a few things that we need before we can proceed with the official acceptance of your study.
We thank you for the handling of our manuscript through the reviewing process, please find here our response to the editorial comments and Reviewer #2 minor points. As requested by Reviewer #2 and by the editor we now removed Figure 4A,B and Appendix Figure S2G. We removed the figure legends of those figures and edited the text accordingly. The immunofluorescence protocol was also removed from the material and methods section.

1) Your article currently has 5 figures and will therefore be published in our Reports section, therefore please combine the Results and Discussion section. If you feel that an extended discussion section is essential, you can also resubmit the manuscript with 6 figures (e.g., a separate figure for the model). In this case you can leave the Results and Discussion section separate. As suggested by the editor and because we believe that a separate discussion section is important we moved the model from old Figure 5G to new Figure 6.

2) Please provide up to five keywords: 5 key words were added just after the abstract. ER stress, Endoplasmic reticulum, ERAD, Reflux, Cancer.

3) Please provide Conflict of Interest and Author Contribution paragraphs. The two paragraphs were added after the acknowledgement.
CONFLICT OF INTEREST
The sentence “EC is cofounder of Cell Stress Discoveries Ltd.” has been added

AUTHOR CONTRIBUTIONS
DS, EC and AI designed the experiments. DS performed the experiments with FGC, RP, PJLR and SC. RP and PJLR conducted the work on murine and human tumors. LN and DS conducted and analyzed the mass spectrometry data. DT, RG and TH provided technical support and helped in data analysis. EC and AI conceived the project, supervised the research, and wrote the manuscript with intellectual input and editing from all authors.

4) Please move the Figure legends to the end of the manuscript (after the references) . The figure legends were moved to after the references as requested.

5) Please update the reference format to match the style of EMBO Reports (list the first 10 authors followed by et. al. and put the year in brackets). See also

https://www.embopress.org/page/journal/14693178/authorguide#referencesformat.
The references format was updated and changed as requested.

6) Please update the relevant fields on funding information in our online submission system.
The field on funding information was updated online as requested.

7) Supplementary Information:
- Please change the nomenclature to "Appendix" and "Appendix Figure Sx", "Appendix Table Sx". Please also update the relevant callouts in the manuscript text.
The nomenclature was changed as requested and the text was edited accordingly.
- Please add page numbers to the table of content on the first page.
Page numbers were added on the first page as requested.
- Please move the Supplementary materials and methods and the antibody table to the Material and Methods section of the main manuscript.
Supplementary materials and methods and the antibody table were moved to the main manuscript as requested.
- Legends:
S1B: please define 'NT' and 'T'
The figure legends were edited accordingly. T= Tumor, NT=non-Tumor.
S1E: please define the number and nature of the experiments and the horizontal black line.
The Figure legends were edited accordingly.
S2B: please provide a scale bar
Scale bars were added as requested.
S3A: please define the concentrations for Tm, Tg, and BFA.
The concentrations were defined in the Figure legends of this Figure.
S3D-F: please define the bars and error bars and the number of experiments.
The bars, error bars and the number of experiments were defined as requested.
S3H, I: please define the units for the concentration of Tm etc (100 xx), the number of experiments and the nature of bars and error bars.
The concentrations, the number of experiment and the nature of bars and error bars were defined in the figure legends of this figure.

8) Author checklist/ethics statements:
- The sentence in 4.a appears incomplete ("For all experiments requiring").
We now fixed this in 4.a, this sentence was misplaced.

- Please complete section D - Animal models in the checklist since you report on tumor cell implantation experiments in mice.
We completed the missing section D-Animal models in the checklist as requested.
- Please add an ethics and approval statement regarding the human tumor samples in the Author checklist and in the methods section of the manuscript.
The ethics and approval statement were added in the checklist and in the methods section of the manuscript as requested.

9) Data availability section: Please add a link that resolves to the dataset.

The link was added in the Data availability section, please note that the data will be published once the paper in accepted for publication.

10) I attach a document with some comments and edits in the figure legends. You have already replied to most of the comments from our data editors before the manuscript was re-reviewed but some points remain unresolved and I also added a few more items you need to address. Moreover, I took the liberty to suggest some changes to the title and abstract. Please review these.
Thank you for taking the time to edit the abstract and the title. Changes were reviewed and accepted. The rest of the request were all addressed.

11) Finally, EMBO reports papers are accompanied online by A) a short (1-2 sentences) summary of the findings and their significance, B) 2-3 bullet points highlighting key results and C) a synopsis image that is 550x200-600 pixels large (width x height) in .png format. You can either show a model or key data in the synopsis image. Please note that the size is rather small and that text needs to be readable at the final size. Please send us this information along with the revised manuscript.
A) short (1-2 Sentences) summary:
"Cancer cells activates Endoplasmic Reticulum (ER) stress which then cause a subset of ER proteins to escape to the cytosol. In the cytosol they bind and inhibit key signaling pathways to increase cancer cell fitness"
B) 2-3 Bullet points

- ER stress mediated protein reflux is a **conserved ER surveillance mechanism from yeast to mammals** that plays a physiological role to **relieve the ER from its contents upon ER stress.**
- ER refluxed proteins **gain new functions once they are in the cytosol**.
- It has a pathophysiological role that we identified which is **"non-genetic inhibition of p53 signaling"** through the reflux of the PDI-like AGR2 that binds to p53 protein an inhibits its activity.

C) synopsis image provided.

Reviewers' comments:

Referee #1:
I went through the point-by-point response to my original comments and through the revised manuscript. As far as I can see, the authors have addressed all my initial comments and I have no further comments or suggestions.
We thank this reviewer for his comments through the reviewing process and his positive and constructive comments on our manuscript.

Referee #2:
The authors have performed additional experiments to address most of my concerns. In particular, they added proteinase K experiments and redox-sensitive probes, trying to strengthen the argument that the observed phenomenon is not due to membrane

rupture/leakage. In general, I find these efforts acceptable. There are still a few minor comments for the authors to consider and revise:
We thank reviewer #2 for his comments through the reviewing process and his positive and constructive comments on our manuscript. We now have addresses his comments as shown here:

1) When calnexin was digested by proteinase K, a lower band would often appear, if the authors saw this band, it would be helpful to include it in the blot. The disappearance of the full-length band should correlate with the accumulation of the lower band.
In this experiment we used a version of antibodies against calnexin (CANX) that recognizes the cytosolic part of the protein for this when this side is degraded by proteinase-k we were unable to see the protein left in the ER lumenal side.

2) Many text labels in the figures are too close to lines or blots. For examle, in Fig 1B, the "NT" and "T" are stepping on the blot below. please adjust accordingly.
We thank the reviewer for this comment, we have now fixed those issues with the figure labeling.

3) The label in S2E is misplaced, please fix.
We now fixed the misplaced label in this figure.

4) I still find Fig. 4A,B problematic. The relocation of the PDIA3 is very difficult to see. The Pearson analysis used rely heavily on the levels of these fluorescent signals. For example, ER inducer suppose to increase the levels of calnexin and PDI, but they are induced to different extents, it could easily influence the coefficients. Furthermore, there is an apparent nuclear import of PI3K upon stress. The reduction of the coefficients could be due to the reduction of cytosolic PI3K, instead of the increase of cytosolic PDIA3. Please consider deleting this part as previously suggested.
Please refer to our response to the editor above, Addressed above

Referee #3:
This revision has largely addressed most of my previous concerns.
We thank this reviewer for his comments through the reviewing process and his positive and constructive comments on our manuscript. As requested by Reviewer #2 and by the editor we now removed Figure 4 A,B and Appendix Figure S2G. We removed the figure legends of those figures and edited the text accordingly. The immunofluorescence protocol was also removed from the material and methods section.

2nd Revision - Editorial Decision 17th Feb 2021

Aeid Igbaria
INSERM U1242
France

Dear Dr. Igbaria,

Thank you for implementing the last minor changes. I have uploaded the revised manuscript files you sent and am now very pleased to accept your manuscript for publication in the next available issue of EMBO reports. Thank you for your contribution to our journal.

At the end of this email I include important information about how to proceed. Please ensure that you take the time to read the information and complete and return the necessary forms to allow us to publish your manuscript as quickly as possible.

As part of the EMBO publication's Transparent Editorial Process, EMBO reports publishes online a Review Process File to accompany accepted manuscripts. As you are aware, this File will be published in conjunction with your paper and will include the referee reports, your point-by-point response and all pertinent correspondence relating to the manuscript.

If you do NOT want this File to be published, please inform the editorial office within 2 days, if you have not done so already, otherwise the File will be published by default [contact: emboreports@embo.org]. If you do opt out, the Review Process File link will point to the following statement: "No Review Process File is available with this article, as the authors have chosen not to make the review process public in this case."

Should you be planning a Press Release on your article, please get in contact with emboreports@wiley.com as early as possible, in order to coordinate publication and release dates.

Thank you again for your contribution to EMBO reports and congratulations on a successful publication. Please consider us again in the future for your most exciting work.

Yours sincerely,

Martina Rembold, PhD
Senior Editor
EMBO reports

****

THINGS TO DO NOW:

You will receive proofs by e-mail approximately 2-3 weeks after all relevant files have been sent to our Production Office; you should return your corrections within 2 days of receiving the proofs.

Please inform us if there is likely to be any difficulty in reaching you at the above address at that

time. Failure to meet our deadlines may result in a delay of publication, or publication without your corrections.

All further communications concerning your paper should quote reference number EMBOR-2020-51412V3 and be addressed to emboreports@wiley.com.

Should you be planning a Press Release on your article, please get in contact with emboreports@wiley.com as early as possible, in order to coordinate publication and release dates.

EMBO PRESS

YOU MUST COMPLETE ALL CELLS WITH A PINK BACKGROUND ↓

PLEASE NOTE THAT THIS CHECKLIST WILL BE PUBLISHED ALONGSIDE YOUR PAPER

Corresponding Author Name: Aeid Igbaria and Eric Chevet

Journal Submitted to: EMBO reports
Manuscript Number: EMBOR-2020-51412V2

USEFUL LINKS FOR COMPLETING THIS FORM

http://www.antibodypedia.com
http://1degreebio.org
http://www.equator-network.org/reporting-guidelines/improving-bioscience-research-repor
http://grants.nih.gov/grants/olaw/olaw.htm
http://www.mrc.ac.uk/Ourresearch/Ethicsresearchguidance/Useofanimals/index.htm
http://ClinicalTrials.gov
http://www.consort-statement.org
http://www.consort-statement.org/checklists/view/32-consort/66-title
http://www.equator-network.org/reporting-guidelines/reporting-recommendations-for-tumo
http://datadryad.org
http://figshare.com
http://www.ncbi.nlm.nih.gov/gap
http://www.ebi.ac.uk/ega
http://biomodels.net/
http://biomodels.net/miriam/
http://jjj.biochem.sun.ac.za
http://oba.od.nih.gov/biosecurity/biosecurity_documents.html
http://www.selectagents.gov/

Reporting Checklist For Life Sciences Articles (Rev. June 2017)

This checklist is used to ensure good reporting standards and to improve the reproducibility of published results. These guidelines are consistent with the Principles and Guidelines for Reporting Preclinical Research issued by the NIH in 2014. Please follow the journal's authorship guidelines in preparing your manuscript.

A- Figures
1. Data
The data shown in figures should satisfy the following conditions:

- the data were obtained and processed according to the field's best practice and are presented to reflect the results of the experiments in an accurate and unbiased manner.
- figure panels include only data points, measurements or observations that can be compared to each other in a scientifically meaningful way.
- graphs include clearly labeled error bars for independent experiments and sample sizes. Unless justified, error bars should not be shown for technical replicates.
- if n< 5, the individual data points from each experiment should be plotted and any statistical test employed should be justified
- Source Data should be included to report the data underlying graphs. Please follow the guidelines set out in the author ship guidelines on Data Presentation.

2. Captions

Each figure caption should contain the following information, for each panel where they are relevant:

- a specification of the experimental system investigated (eg cell line, species name).
- the assay(s) and method(s) used to carry out the reported observations and measurements
- an explicit mention of the biological and chemical entity(ies) that are being measured.
- an explicit mention of the biological and chemical entity(ies) that are altered/varied/perturbed in a controlled manner.

- the exact sample size (n) for each experimental group/condition, given as a number, not a range;
- a description of the sample collection allowing the reader to understand whether the samples represent technical or biological replicates (including how many animals, litters, cultures, etc.).
- a statement of how many times the experiment shown was independently replicated in the laboratory.
- definitions of statistical methods and measures:
  - common tests, such as t-test (please specify whether paired vs. unpaired), simple χ2 tests, Wilcoxon and Mann-Whitney tests, can be unambiguously identified by name only, but more complex techniques should be described in the methods section;
  - are tests one-sided or two-sided?
  - are there adjustments for multiple comparisons?
  - exact statistical test results, e.g., P values = x but not P values < x;
  - definition of 'center values' as median or average;
  - definition of error bars as s.d. or s.e.m.

Any descriptions too long for the figure legend should be included in the methods section and/or with the source data.

In the pink boxes below, please ensure that the answers to the following questions are reported in the manuscript itself. Every question should be answered. If the question is not relevant to your research, please write NA (non applicable). We encourage you to include a specific subsection in the methods section for statistics, reagents, animal models and human subjects.

B- Statistics and general methods

Please fill out these boxes ↓ (Do not worry if you cannot see all your text once you press return)

1.a. How was the sample size chosen to ensure adequate power to detect a pre-specified effect size?	by default all experiments carried out are with a minimum of n=3
1.b. For animal studies, include a statement about sample size estimate even if no statistical methods were used.	n=6 for analysis of the GBM tumors.
2. Describe inclusion/exclusion criteria if samples or animals were excluded from the analysis. Were the criteria pre-established?	No exclusion performed
3. Were any steps taken to minimize the effects of subjective bias when allocating animals/samples to treatment (e.g. randomization procedure)? If yes, please describe.	No treatment to animal experiments
For animal studies, include a statement about randomization even if no randomization was used.	Random selection of mice in both GBM tumors (GL261 and NSG)
4.a. Were any steps taken to minimize the effects of subjective bias during group allocation or/and when assessing results (e.g. blinding of the investigator)? If yes please describe.	N/A
4.b. For animal studies, include a statement about blinding even if no blinding was done	N/A
5. For every figure, are statistical tests justified as appropriate?	YES
Do the data meet the assumptions of the tests (e.g., normal distribution)? Describe any methods used to assess it.	YES, included in the GrapPad software Prism
Is there an estimate of variation within each group of data?	YES, included in the GrapPad software Prism

Is the variance similar between the groups that are being statistically compared?	YES, included in the GrapPad software Prism

C- Reagents

6. To show that antibodies were profiled for use in the system under study (assay and species), provide a citation, catalog number and/or clone number, supplementary information or reference to an antibody validation profile. e.g., Antibodypedia (see link list at top right), 1DegreeBio (see link list at top right).	This is provided in the materials and methods section
7. Identify the source of cell lines and report if they were recently authenticated (e.g., by STR profiling) and tested for mycoplasma contamination.	This is provided in the materials and methods section

* for all hyperlinks, please see the table at the top right of the document

D- Animal Models

8. Report species, strain, gender, age of animals and genetic modification status where applicable. Please detail housing and husbandry conditions and the source of animals.	Animl work was done as follows and as described in material and methods section under"Mouse Work": Tumor cell orthotopic Implantation – Tumor cells (GL261) were implanted in the brain of immunocompetent C57BL/6rJ, 8 weeks old male mice (Janvier, Laval, France) and tumor cells (U87) were implanted in the brain of immunodeficient mice NSG (NOD.Cg-Prkdcscid Il2rgtm1Wjl/SzJ). Mice were purchased from Charles River Laboratories (Wilmington, MA), 8 weeks old male mice (Janvier, Laval, France). Animal housing was carried ensuring the breeding and the daily monitoring of the animals in the best conditions of well-being according to the law and the rule of 3R (Reduce-Refine-Replace).
9. For experiments involving live vertebrates, include a statement of compliance with ethical regulations and identify the committee(s) approving the experiments.	All animal procedures met the European Community Directive guidelines (Agreement B35-238-40 Biosit Rennes, France/ No DIR 13480) and were approved by the local ethics committee and ensuring the breeding and the daily monitoring of the animals in the best conditions of well-being according to the law and the rule of 3R (Reduce-Refine-Replace).
10. We recommend consulting the ARRIVE guidelines (see link list at top right) (PLoS Biol. 8(6), e1000412, 2010) to ensure that other relevant aspects of animal studies are adequately reported. See author guidelines, under 'Reporting Guidelines'. See also: NIH (see link list at top right) and MRC (see link list at top right) recommendations. Please confirm compliance.	We confirm compliance

E- Human Subjects

11. Identify the committee(s) approving the study protocol.	All human samples used for the analyses shown in this manuscript were provided by the Centre de Ressources Biologiques (CRB) Santé of Rennes BB-0033-0005. Informed consent was obtained in accordance with the French legislation under the auspices of French National autorities.
12. Include a statement confirming that informed consent was obtained from all subjects and that the experiments conformed to the principles set out in the WMA Declaration of Helsinki and the Department of Health and Human Services Belmont Report.	OK
13. For publication of patient photos, include a statement confirming that consent to publish was obtained.	N/A
14. Report any restrictions on the availability (and/or on the use) of human data or samples.	N/A
15. Report the clinical trial registration number (at ClinicalTrials.gov or equivalent), where applicable.	N/A
16. For phase II and III randomized controlled trials, please refer to the CONSORT flow diagram (see link list at top right) and submit the CONSORT checklist (see link list at top right) with your submission. See author guidelines, under 'Reporting Guidelines'. Please confirm you have submitted this list.	N/A
17. For tumor marker prognostic studies, we recommend that you follow the REMARK reporting guidelines (see link list at top right). See author guidelines, under 'Reporting Guidelines'. Please confirm you have followed these guidelines.	N/A

F- Data Accessibility

18: Provide a "Data Availability" section at the end of the Materials & Methods, listing the accession codes for data generated in this study and deposited in a public database (e.g. RNA-Seq data: Gene Expression Omnibus GSE39462, Proteomics data: PRIDE PXD000208 etc.) Please refer to our author guidelines for 'Data Deposition'.<br><br>Data deposition in a public repository is mandatory for:<br>a. Protein, DNA and RNA sequences<br>b. Macromolecular structures<br>c. Crystallographic data for small molecules<br>d. Functional genomics data<br>e. Proteomics and molecular interactions	N/A
19. Deposition is strongly recommended for any datasets that are central and integral to the study; please consider the journal's data policy. If no structured public repository exists for a given data type, we encourage the provision of datasets in the manuscript as a Supplementary Document (see author guidelines under 'Expanded View' or in unstructured repositories such as Dryad (see link list at top right) or Figshare (see link list at top right).	N/A
20. Access to human clinical and genomic datasets should be provided with as few restrictions as possible while respecting ethical obligations to the patients and relevant medical and legal issues. If practically possible and compatible with the individual consent agreement used in the study, such data should be deposited in one of the major public access-controlled repositories such as dbGAP (see link list at top right) or EGA (see link list at top right).	N/A
21. Computational models that are central and integral to a study should be shared without restrictions and provided in a machine-readable form. The relevant accession numbers or links should be provided. When possible, standardized format (SBML, CellML) should be used instead of scripts (e.g. MATLAB). Authors are strongly encouraged to follow the MIRIAM guidelines (see link list at top right) and deposit their model in a public database such as Biomodels (see link list at top right) or JWS Online (see link list at top right). If computer source code is provided with the paper, it should be deposited in a public repository or included in supplementary information.	N/A

G- Dual use research of concern

22. Could your study fall under dual use research restrictions? Please check biosecurity documents (see link list at top right) and list of select agents and toxins (APHIS/CDC) (see link list at top right). According to our biosecurity guidelines, provide a statement only if it could.	No
